# Enhancing thermoelectric properties of single-walled carbon nanotubes using halide compounds at room temperature and above

**DOI:** 10.1038/s41598-021-88079-w

**Published:** 2021-04-21

**Authors:** Bogumiła Kumanek, Grzegorz Stando, Paweł Stando, Karolina Matuszek, Karolina Z. Milowska, Maciej Krzywiecki, Marta Gryglas-Borysiewicz, Zuzanna Ogorzałek, Mike C. Payne, Douglas MacFarlane, Dawid Janas

**Affiliations:** 1 Department of Organic Chemistry, Bioorganic Chemistry and Biotechnology, Silesian University of Technology, B. Krzywoustego 4, 44-100 Gliwice, Poland; 2 School of Chemistry, Monash University, Clayton, VIC 3800 Australia; 3 TCM Group, Cavendish Laboratory, University of Cambridge, 19 JJ Thomson Avenue, Cambridge, CB3 0HE UK; 4 Institute of Physics-CSE, Silesian University of Technology, Konarskiego 22B, 44-100, Gliwice, Poland; 5 Faculty of Physics, University of Warsaw, Pasteura 5, 02-093 Warsaw, Poland

**Keywords:** Materials science, Nanoscience and technology

## Abstract

Carbon nanotubes (CNTs) are materials with exceptional electrical, thermal, mechanical, and optical properties. Ever since it was demonstrated that they also possess interesting thermoelectric properties, they have been considered a promising solution for thermal energy harvesting. In this study, we present a simple method to enhance their performance. For this purpose, thin films obtained from high-quality single-walled CNTs (SWCNTs) were doped with a spectrum of inorganic and organic halide compounds. We studied how incorporating various halide species affects the electrical conductivity, the Seebeck coefficient, and the Power Factor. Since thermoelectric devices operate under non-ambient conditions, we also evaluated these materials' performance at elevated temperatures. Our research shows that appropriate dopant selection can result in almost fivefold improvement to the Power Factor compared to the pristine material. We also demonstrate that the chemical potential of the starting CNT network determines its properties, which is important for deciphering the true impact of chemical and physical functionalization of such ensembles.

## Introduction

With demand for energy rising year by year, it is necessary to look for solutions that will allow for the best possible energy use at minimum losses. Unfortunately, at present, these losses are very significant. For instance, many engines' energy efficiency used in everyday life does not exceed 55%^1^. Recently, it has become clear that this inefficiency can be approached differently. Waste energy generated in the form of heat can be recycled using the so-called thermoelectric devices, which can turn a fraction of thermal energy back into useful electrical energy. To make this approach viable, the materials selected should ideally be easy to process, non-toxic, and inexpensive.

Unfortunately, the materials known to demonstrate high thermoelectric efficiencies, such as Bi_2_Te_3_^2,3^, Sb_2_Te_3_^4–6^_,_ or PbTe^2,5,7^, do not meet one or more of these conditions. The solution to that problem can be to use carbon nanotubes (CNTs) to satisfy the aforementioned criteria better. Not only do they demonstrate encouraging electrical^8,9^, thermal^10,11^, and mechanical characteristics^12,13^, but also, most importantly, notable thermoelectric properties^14^. For instance, the Seebeck coefficient, which is indicative of how well the material induces thermoelectric voltage per unit temperature difference, reaches up to 230 μV/K^15^ for individual semiconducting single-walled CNTs. However, when they are combined to form networks, their Seebeck coefficient is usually on the order of 50 μV/K. Despite that problem, caused mostly by contact resistance^16^, the thermoelectric capabilities of these materials remain promising.

Another fundamental property of a thermoelectric device is how well it conducts electric current. Since the material should have the highest possible electrical conductivity, any means to boost this property is welcome. The simplest way to tackle this challenge is to introduce additional species to the CNT ensemble, which can readily shift the Fermi level by injecting electrons or holes into the system, giving n- and p-doping, respectively. Doping can be achieved in various ways, such as embedding doping atoms in the structure of the CNT lattice^17,18^, encapsulation of metals or compounds inside the CNT^19^, or, most straightforwardly, simple adsorption of doping agents onto the surface of CNTs^20,21^.

In the studies carried out so far on the doping of CNTs to improve their electrical and thermoelectric properties, various agents have been applied, both inorganic and organic. One of the highest values of electrical conductivity resulting from doping were reported by Zhao et al*.*^22^, who obtained iodine-doped CNT fibers, for which electrical conductivity was as high as 6.67 · 10^6^ S/m. Recently, Nonoguchi and et al*.*^23^ verified the impact of many organic doping agents containing nitrogen and phosphorus on thermoelectric properties. In these studies, significant changes to the Seebeck coefficient values were noted, but the highest Power Factor (PF, *defined below*) values did not exceed 26 µW/m·K^2^. Moreover, Ryu et al*.*^24^ demonstrated that doping CNTs with HSO_3_Cl significantly improved the value of the PF. Research conducted so far has indicated clearly that halogen compounds, or compounds containing a halogen atom, belong to one of the best groups of doping agents and improve electrical and thermoelectric properties effectively^25,26^. The effectiveness of the halides results from their atomic structure, which determines how the dopant will interact with the host material to be doped^22,27^. Table S1 gives an overview of the dopants containing a halide atom that have been used to modulate the electrical and thermoelectric properties of CNT ensembles. Enhancements of up to two orders of magnitude have been reported^22–24,28–34^. To gauge the suitability of a material for thermoelectric applications, the so-called Power Factor (PF) is commonly determined for various materials, using the following formula:

1 $${\text{PF}} = {\text{S}}^{2} \cdot \sigma$$

where PF—Power Factor [µW/m·K^2^], S—Seebeck coefficient [μV/K], σ—electrical conductivity [S/m].

Ideally, a good thermoelectric material should demonstrate both a high Seebeck coefficient and high electrical conductivity. As the present state of the art shows, the effect of halide doping on the value of both Seebeck coefficient and Power Factor has not been studied extensively, which attracted our attention. In this study, we examined the influence of a broad spectrum of compounds incorporating halide atoms on the electrical conductivity of CNT networks. After analyzing the results, we selected ten that best improved electrical conductivity and subjected them to a more detailed characterization. For these, we measured the electrical conductivity and Seebeck coefficient values at elevated temperatures (r.t., 40 °C, 70 °C, and 100 °C). From the obtained values, we calculated the values of PF [based on Eq. (1)] to evaluate which doping agent gives the most promising results across a chosen temperature range. This is important in the context of the potential application of such formulations for the recovery of waste energy in the form of heat from e.g. car engines, the exterior temperature of which is at ca. 85–95 °C^35^. Lastly, we conducted spectroscopy and computation to elucidate the underlying mechanisms responsible for enhancing CNT films’ performance.

## Results and discussion

**Figure 1:**
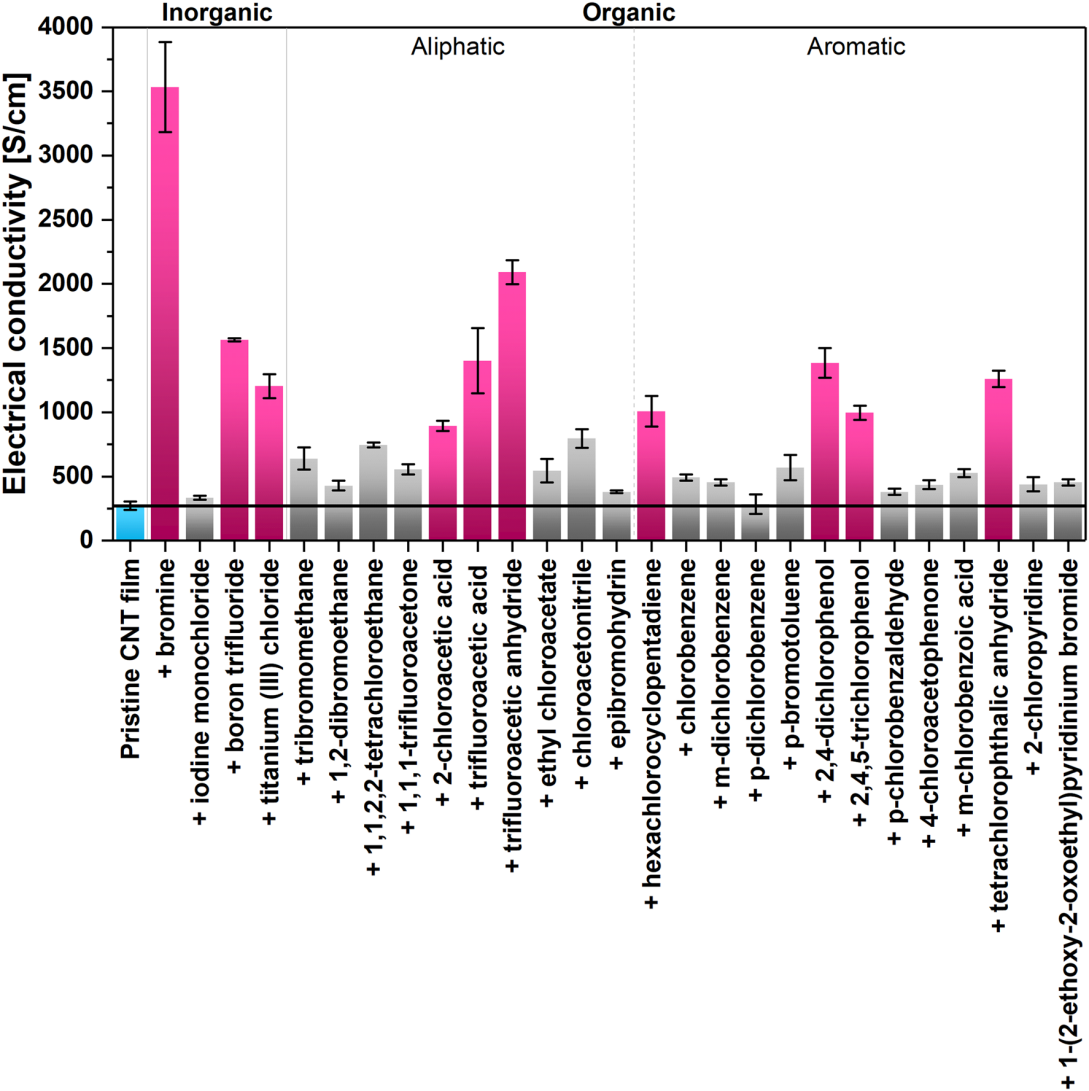
Electrical conductivity at room temperature of each CNT film sample.

We initiated the study by screening the electrical characteristics of the selected materials, which are shown in Fig. 1.

Films made from undoped SWCNTs had an electric conductivity of 272 ± 33 S/cm at room temperature. Various conductivity values of such networks of CNTs can be found in the literature. The values mainly depend on the type of CNTs, their length, alignment, packing degree, chirality distribution, and degree of structural perfection of the material^9,36^. Consequently, the electrical conductivity of such single-walled CNT films commonly spans many orders of magnitude: from 10^1^ S/m to 5 · 10^5^ S/m^23,37,38^. The electrical conductivity of the initial undoped material we measured (2.72 · 10^4^ S/m) was competitive with the state-of-the-art, which encouraged us to probe how much this conductivity can be enhanced by doping.

Our studies showed that doping CNTs with compounds containing a halogen atom improves their electrical conductivity substantially. The increase in the value of electrical conductivity, depending on the dopant, ranges from a few percent to as much as 1200%. The smallest effect was found for simple substituted hydrocarbons and aromatic compounds functionalized with halide atoms, excluding halide derivatives of phenol. The strongest influence of doping on electrical properties, on the other hand, was recorded when 0.1 M bromine solution in water was used. The addition of bromine gave rise to an enhancement of the material's electrical conductivity from 272 ± 33 to 3533 ± 352 S/cm. Acetic acid derivatives, phenol derivatives, and acid anhydrides containing a halide atom also significantly impacted electrical properties. In particular, addition of 2,4-dichlorophenol, 2,4,5-trichlorophenol, trifluoroacetic acid and trifluoroacetic anhydride resulted in electrical conductivity increases of 407%, 264%, 415%, and 669% (reaching 1385 ± 115 S/cm, 996 ± 54 S/cm, 1403 ± 254 S/cm and 2091 ± 92 S/cm), respectively. Notable improvements were also observed in the case of boron trifluoride, titanium chloride, 2-chloroacetic acid, hexachlorocyclopentadiene, and tetrachlorophtalic anhydride. The compounds described above that had the strongest influence on the value of electrical conductivity of CNT films (highlighted in pink in Fig. 1) were subject to additional measurements: Raman analysis, thermogravimetric analysis as well as characterization of electrical conductivity and Seebeck coefficient values at r.t., 40 °C, 70 °C, and 100 °C.

To fully understand the nature of the changes induced by adding a dopant, it is necessary first to establish the impact of its presence on the structure and electronic properties of the CNT network. Raman spectroscopy enabled us to determine that no chemical modification occurs upon introducing the doping species into the CNT network. The I_D_/I_G_ ratio, which is commonly used for determining the fraction of carbon atoms with sp^3^ hybridization (indicative of impurities/functionalization) to carbon atoms with sp^2^ hybridization (representative of graphitic lattice), respectively, remained essentially unchanged (Table 1—values, Fig. S1—corresponding spectra). It should be noted that the I_D_/I_G_ ratios of both the pristine material and the doped samples were very low and did not exceed 0.042. Slight deviations are within the expected statistical error for such a low value of the I_D_/I_G_ ratios. Therefore, we concluded that chemical modification of the CNTs by these doping species was unlikely under these conditions.

**Table 1:** Analysis of doped CNT films by Raman spectroscopy.

No.	Sample	G peak maximum (cm^−1^)	Shift (cm^−1^)	I_D_/I_G_ (–)
	Pristine CNT film	1590.0	0	0.015
1	+ bromine	1598.5	8.5	0.046
2	+ boron trifluoride	1592.5	2.5	0.014
3	+ titanium(III) chloride	1592.3	2.3	0.018
4	+ 2-chloroacetic acid	1592.9	2.9	0.029
5	+ trifluoroacetic acid	1591.0	1	0.042
6	+ trifluoroacetic acid anhydride	1591.0	1	0.038
7	+ hexachlorocyclopentadiene	1592.3	2.3	0.026
8	+ 2,4-dichlorophenol	1591.0	1	0.014
9	+ 2,4,5-trichlorophenol	1591.0	1	0.035
10	+ tetrachlorophtalic anhydride	1591.0	1	0.025

Important information about doping is also provided by the location of the G peak maximum. For the pristine CNT material, the maximum was observed at 1590 cm^−1^. All doping agents caused a shift of the G peak maximum towards higher wavenumbers, which means that the Fermi level was shifted downwards by the doping process. The observed blue shift indicates p-doping of the material^39^. The results show that the strongest impact on the electronic properties of the material resulted from the addition of bromine (G peak maximum position shifted by 8.5 cm^−1^). This was expected on the basis of the very electrophilic nature of these species. The shift in the case of other dopants was smaller, spanning from 1 to 2.9 cm^−1^. It has to be noted that the host material is naturally p-doped by oxygen present in the ambient^40^. Thus, it is challenging to induce further blue-shift to the Raman spectra.

**Figure 2:**
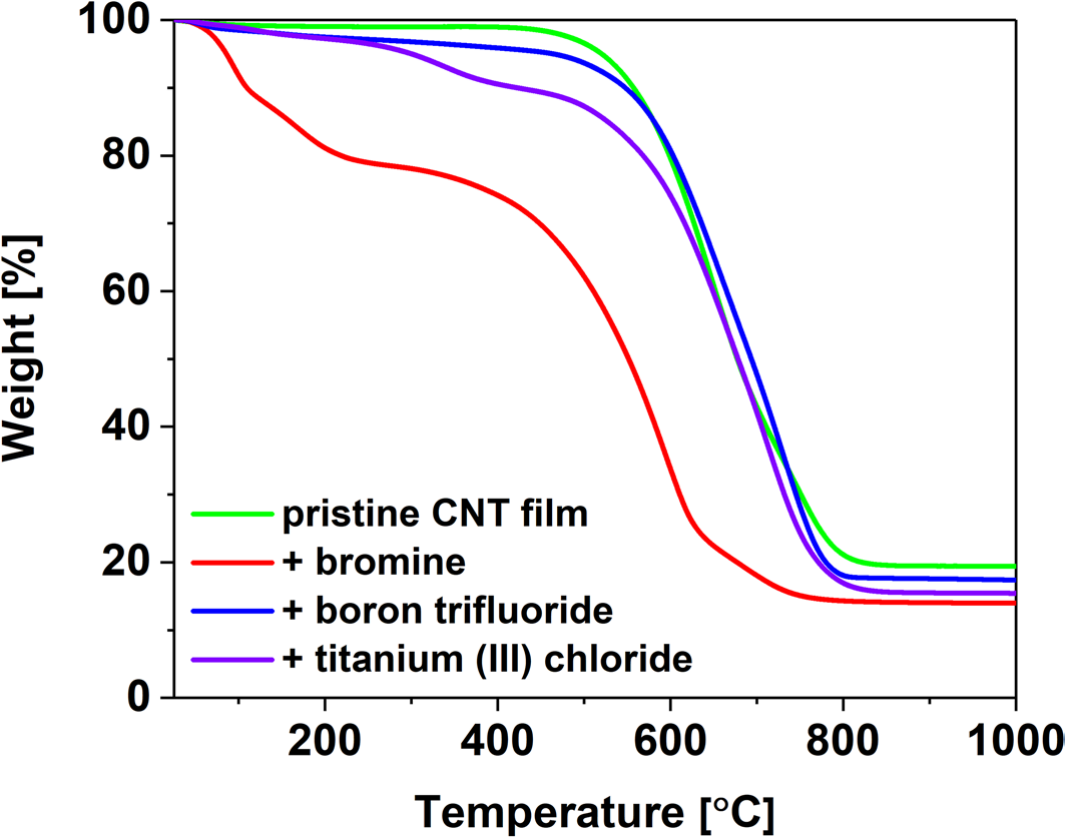
Thermograms of a CNT film before and after doping with inorganic halide compounds.

**Figure 3:**
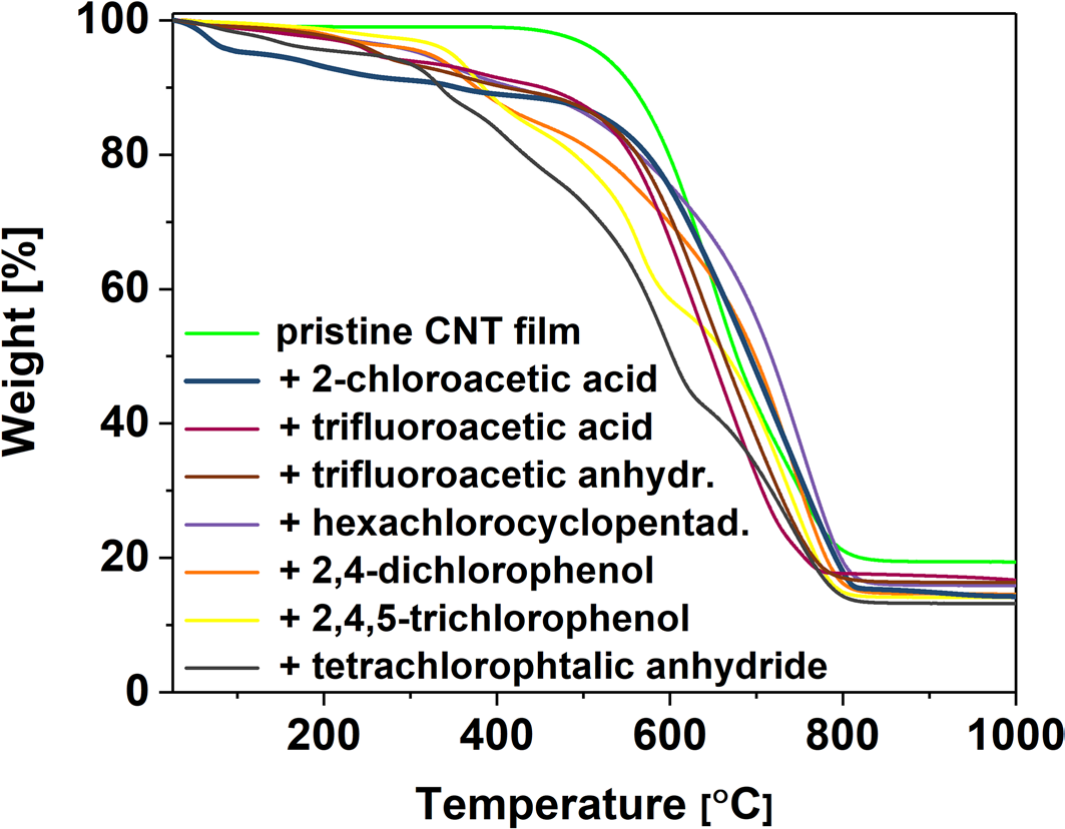
Thermograms of a pristine CNT film and CNT films after doping with organic halide compounds.

Next, we wanted to study the stability of the doping as a function of temperature. For this purpose, thermogravimetric analysis was carried out for selected films, the results of which are presented in Fig. 2 for inorganic dopants and Fig. 3 for organic ones. In addition, we provide a summary of the results of the analysis of the first derivatives of the mass loss curves (Table S2) with example first derivative plots for the pristine CNT film (Fig. S2) and the CNT film doped with 2,4,6,7-tetrachlorophthalitic anhydride (Fig. S3) in the supplementary information.

The pristine CNT film shows very good thermal stability up to about 434 °C, above which it undergoes thermal degradation with the maximum rate at T_max_ = 651 °C. Upon doping, the thermal characteristics of CNT networks change significantly. In the case of the materials doped with inorganic compounds, we observe that the maximum degradation rate occurs at higher temperatures (Fig. 2)—T_max_ of 679 °C, 729 °C and 711 °C for bromine, boron trifluoride and titanium (III) chloride, respectively. However, we also notice the appearance of signals indicating weight loss at lower temperatures corresponding to the loss of the doping agent. In the case of bromine, it can be seen that its removal occurs already in a low-temperature regime. On the other hand, materials doped with titanium (III) chloride or boron trifluoride exhibited much higher thermal stability.

In the case of organic dopants (Fig. 3), which generally have a higher boiling point (hexachlorocyclopendadiene, 2,4-dichlorophenol, 2,4,5-trichlorophenol, 2-chloroacetic acid), the temperature of the maximum degradation rate of doped CNT films is about 740 °C. Furthermore, the first signals of significant changes in mass coincide with their boiling point or the expected decomposition temperature. In the case of materials doped with compounds with lower boiling temperatures, the maximum rate of mass loss has been recorded at about 640 °C. The first signs of material degradation are similar to the boiling temperatures typical for these substances. However, signals at higher temperatures can also be observed, which indicates that the entire substance does not evaporate/decompose immediately. We suspect that such long timescales result from the entrapment of some of these species in the CNT network. Both in the cases of inorganic and organic dopants, the thermal stability of the host material was changed after their addition.

Thermal degradation is a complex problem to analyze. First of all, thermogravimetric analysis is a kinetic process. The results are very much dependent on the selected heating rate^41^, which ideally should not outpace the analyte's decomposition kinetics. Unfortunately, this condition is hard to satisfy when different samples of radically different chemical compositions are analyzed. Secondly, the addition of some species is very likely to cause changes in the doped material, which can also influence the course of thermal degradation. For example, densification of the material can affect the dynamics of the oxidation process^41^. Lastly, many of these species themselves have complex degradation pathways that extend to high-temperature regimes. All these phenomena combined have an impact on the shapes of the thermograms.

**Figure 4:**
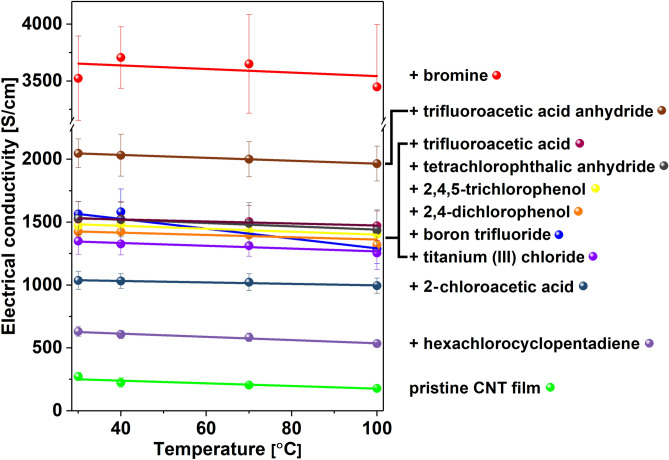
Electrical conductivity at different temperatures for a pristine CNT film and CNT films after doping with halide compounds. Lines were added to guide the eye through a selected group of results.

As the next step, we decided to evaluate the electrical conductivity of the material as a function of temperature (Fig. 4). For a pristine CNT film, the conductivity decreased with increasing temperature. This behavior is typical for the materials that exhibit predominantly metallic character and results from the charge scattering effect. The higher the temperature, the more notable the phenomenon, as expected. Similarly to a pristine CNT film, it can be seen that the conductivity of the doped materials decreases slightly with temperature. Nevertheless, within the explored temperature range, no major decrease of conductivity is observed, demonstrating that the selected doping agents are relatively stable under these conditions.

The only exception to these results comes from the experiments with bromine. In the case of the bromine-doped material, the results obtained by us are characterized by large standard deviations. This is due to the fact that bromine is very volatile and the pristine CNT film doped with bromine is not as stable as others, which can be very clearly seen in the provided thermogram (Fig. 2).

**Figure 5:**
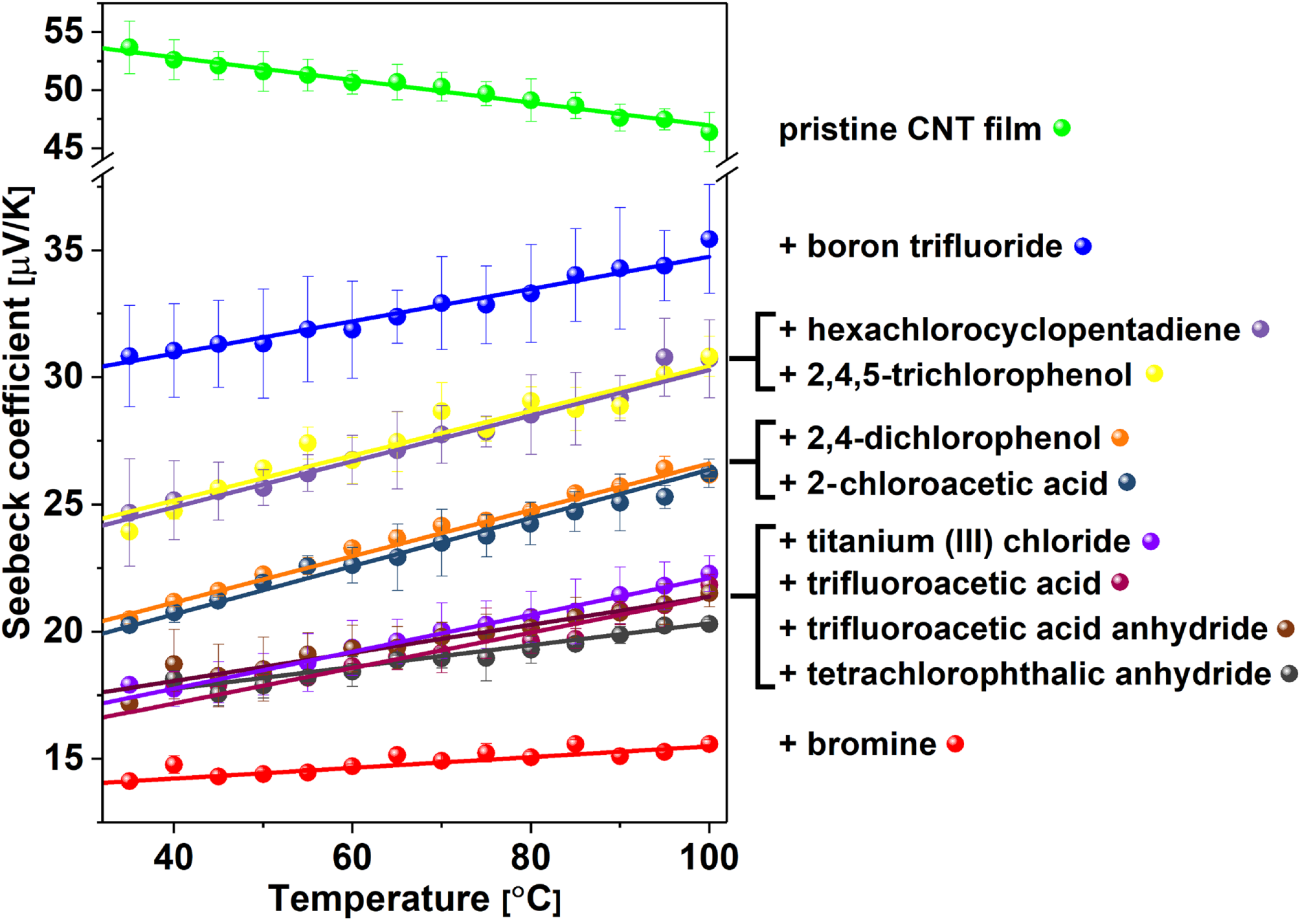
Seebeck coefficient at different temperatures for a pristine CNT film and CNT films after doping with halide compounds. Lines were added to guide the eye through a selected group of results.

At this point, we moved on to the evaluation of the thermoelectric performance of these materials. The changes in the Seebeck coefficient's value as a function of temperature, compared with the pristine material, are listed in Fig. 5. Regarding the results at room temperature, the Seebeck coefficient's highest value was recorded for a pure CNT film, and it was 54 ± 3 μV/K. In the case of doping with bromine, which caused the largest improvement in electrical conductivity, it also gave the lowest Seebeck coefficient of 14.5 ± 0.35 μV/K. The highest values of Seebeck coefficient at room temperature for the modified CNT networks were recorded for materials doped with BF_3_ (30.5 ± 1.5 μV/K), hexachlorocyclopentadiene (24.5 ± 2.6 μV/K), and 2,4,5-trichlorophenol (24.45 ± 0.1 μV/K). Such changes, where the Seebeck coefficient decreases with increasing electrical conductivity, have already been observed^42^. This is because the capability to generate thermopower is strongly linked to the Fermi level, which is affected by factors such as carrier concentration and the effective mass of the carrier. When introducing a dopant into the pristine material, the carrier mobility/density changes, which results in the modulation of electrical conductivity. If either of these parameters increases, the thermopower quantified by the Seebeck coefficient decreases^43^, which we observed above.

It was observed that with the increase in temperature, the Seebeck coefficient decreases in the pristine material, reaching 46.4 ± 1.7 μV/K at 100 °C, which is a deterioration of 13.7%. On the other hand, in the case of doped materials, the value of the Seebeck coefficient increases by between 5% and 33.3%. The most substantial increase was recorded for chloroacetic acid (33.3%) and 2.4-dichlorophenol (32.5%) doping. A boost of 25% was noted for 2,4,6-trichlorophenol and hexachlorocyclopentadiene compounds. We can conclude that the increase in temperature mitigates the loss of thermopower upon doping at room temperature as observed here, and, also, previously for other low-dimensional architectures^43^.

**Figure 6:**
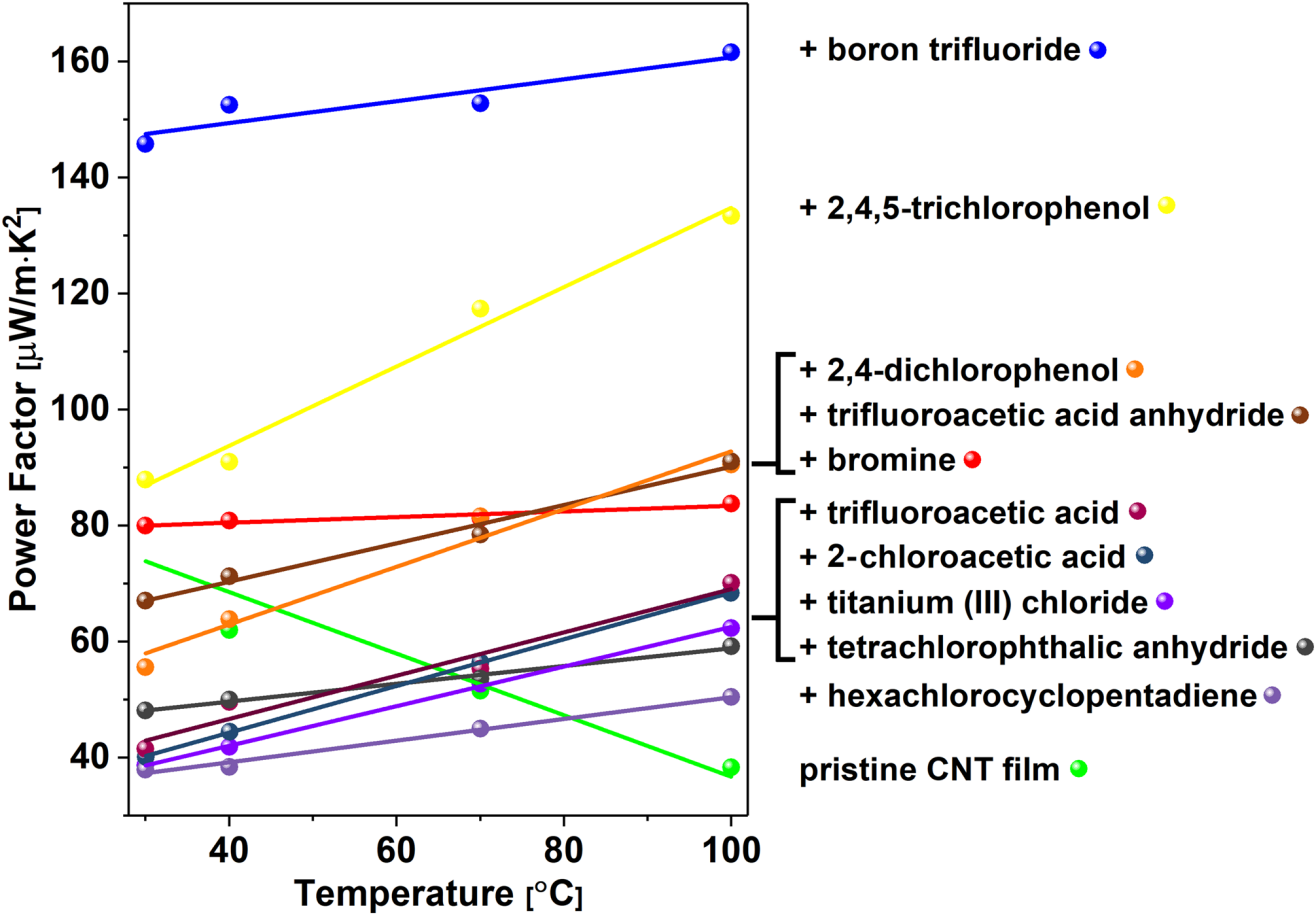
Power Factors at different temperatures for a pristine CNT film and CNT films after doping with halide compounds. Lines were added to guide the eye through a selected group of results.

Since the thermoelectric performance represented by the Power Factor is a function of electrical conductivity and Seebeck coefficient, both of which were measured by us in this study, we investigated the net effect of doping on its value for different materials (Fig. 6). Since the doping caused increases in electrical conductivity but decreases in Seebeck coefficients, we analyzed in detail the combined impact of these two parameters at and above room temperature. At room temperature, only two dopants caused the PF to be higher for the doped material than for pure CNT films (80 µW/m·K^2^). These doping agents were BF_3_ (145.8 µW/m·K^2^) and 2,4,5-trichlorophenol (87.9 µW/m·K^2^). However, as the temperature increases, the beneficial effect of doping becomes even more prominent. The combined substantial improvements of electrical conductivity upon doping and the Seebeck coefficients with temperature in doped CNT networks cause significant enhancement of their thermoelectric performance.

The highest PF value of 161.5 µW/m·K^2^ was recorded at 100 °C for the CNT film doped with BF_3_. This material previously showed an appreciable increase in electrical conductivity, the highest Seebeck coefficient among doped materials, and high thermal stability as gauged by TGA. It is also interesting to note that CNT films doped with 2,4,5-trichlorophenol exhibited a remarkable sensitivity of thermoelectric performance to temperature. An increase in the Power Factor of over 50% was recorded when the temperature of the sample was elevated to 100 °C. At this temperature, the Power Factors of these two formulations (CNTs doped with BF_3_ or 2,4,5-trichlorophenol) are better than those of unmodified CNT films by ca. 420% and 350%, respectively. Since thermoelectric power generation occurs across a temperature gradient, the relevant PF of a thermoelectric module is approximately midway between T_hot_ and T_cold_ at operating steady-state. Considering the presented results, upon doping CNT films with indicated chemical compounds, the PF is clearly enhanced in this temperature regime.

We decided to conduct further experiments to determine the underlying phenomena that gave rise to the above-mentioned enhancements. For these analyses, samples doped with two chemical compounds were selected: Br_2_ and BF_3_. The addition of the former made a remarkable improvement to the electrical conductivity (Fig. 4). The latter, on the other hand, shows a smaller increase to the electrical conductivity (Fig. 4), while it did not suffer a substantial decrease of the Seebeck (Fig. 5) coefficient (which was the case for Br_2_-doped SWCNT films). As a result of the more balanced combined impact of these two factors on the material, the Power Factor of BF_3_-doped CNTs experienced the highest increase (Fig. 6).

**Figure 7:**
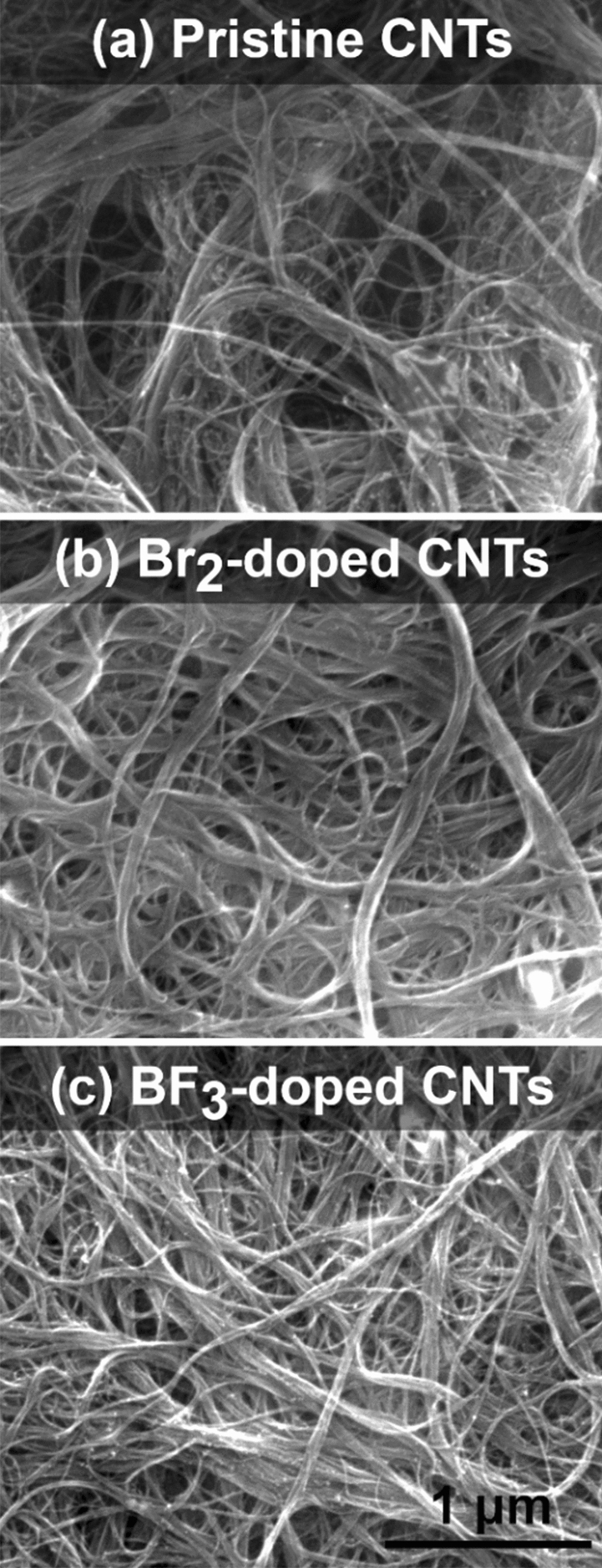
SEM micrographs of (**a**) a pristine CNT film and CNT films after doping with (**b**) Br_2_, and (**c**) BF_3_.

Firstly, the microstructure inspection upon doping of the CNT films with two selected chemical compounds revealed changes introduced by the processing (Fig. 7). The pristine film demonstrated an isotropic distribution of CNTs and their bundles as expected for a network prepared by simple deposition of CNT dispersion onto a substrate. Such materials are typically porous^44,45^, which explains the presence of voids, which are apparent in the micrograph (Fig. 7a). Once the CNT film was doped with either Br_2_ (Fig. 7b) or BF_3_ (Fig. 7c), the degree of packing in the material increased.

Evaporation of the solvent used to deliver the dopants may give rise to capillary densification^46^, thereby reducing the distance between CNTs and their bundles. This, may decrease the contact resistance between SWCNTs and their bundles^47^, and thus minimize the resistance of the whole network, purely because of geometrical reasons. However, since all the halide dopants were introduced in such media, and some of them exhibited virtually no enhancement of electrical conductivity (Fig. 1), this hypothesis can be dismissed. This reasoning is supported by the fact that the Seebeck coefficient remained unaffected when exposed to a neat solvent. In a null experiment conducted by us, simple dipping of a pure CNT film in acetone with subsequent evaporation did not influence the recorded Seebeck coefficient (Fig. S4). Consequently, the densification does not seem to impact the electronic properties of the CNT network, but only slightly improves the charge propagation. To study the phenomena responsible for the observed enhancement, first principles calculations were engaged (vide infra).

**Figure 8:**
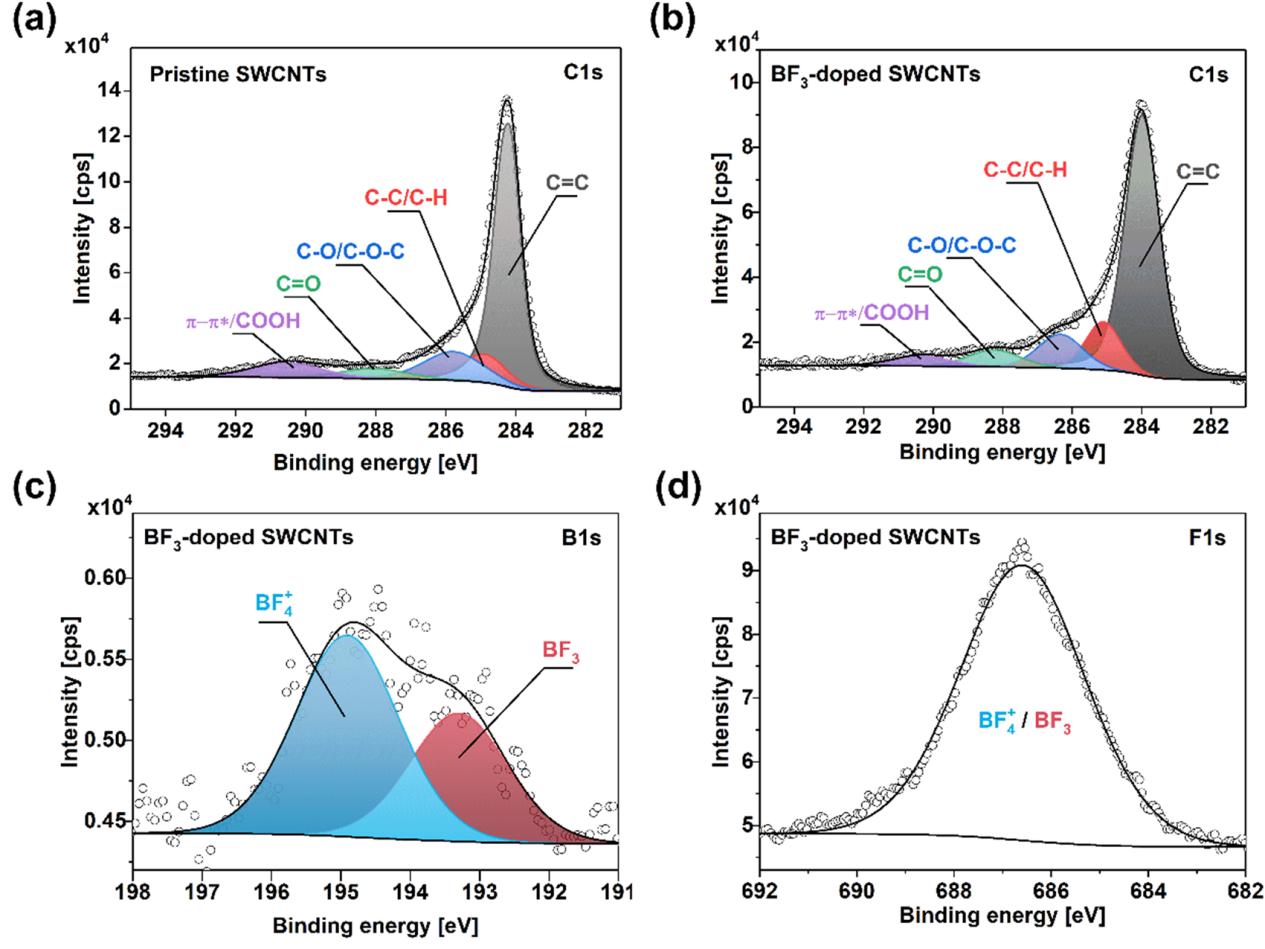
XPS spectra of the (**a**,**b**) neat and (**c**,**d**) Br_2_-doped CNT films. Corresponding (**a**,**c**) C1s, and (**b**,**d**) Br3d areas.

Before that, however, two more characterizations were required to establish appropriate parameters for modelling. Firstly, XPS characterization was employed to prove that physical doping has occurred rather than chemical functionalization (Fig. 8). BF_3_-doped CNT film was used for this analysis because the high volatility of Br_2_ could be problematic in the spectrometer's high-vacuum environment. The results showed that after doping, the CNT films remained well graphitized, and the sp^2^ component was dominant in both cases (Fig. 8a,b). A slight elevation of the C=O and C–O/C–O–C components was ascribed to the residues of methanol used to deliver BF_3_ into the CNT matrix.

The C-B bond could not be discerned in the C1s spectra of the boron-doped CNTs supporting the hypothesis that no chemical modification took place^48,49^. Furthermore, the boron (Fig. 8c) and fluorine (Fig. 8d) signatures were readily detected in the material. Regarding the former, the peak position does not correspond with signatures of C-B bond observed by other researchers at ca. 189 eV^50,51^, so another type of bond configuration must be present. Given that the feature in Fig. 8c is located in the higher energy regime, it could account for bromine in the form of BF_3_ and BF_4_^+^. This is supported by Ahmad and colleagues' results, who observed a B-F component in a comparable region^52^. Furthermore, the peak centered 195 eV could be assigned to BF_4_^+^ species following a similar argument that charged ammonium species are located at higher energies than their corresponding neutral nitrogen functionalities in the N1s area^53–55^. Lastly, the position of the feature in the F1s area is also in line with the literature regarding the expected position for an F-B functionality^52^. Given all this experimental evidence, the results strongly suggest that the doping is physical in nature.

Secondly, it was also essential to estimate the proportion of metallic to semiconducting CNTs in the network for computation. Results of electrical characterization mentioned above revealed qualitatively that the films are predominantly metallic. Absorption spectroscopy estimated the content of metallic and semiconducting CNTs to be ~ 90% and ~ 10%, respectively, following the previously published methodology (Fig. S5)^56,57^.

**Figure 9:**
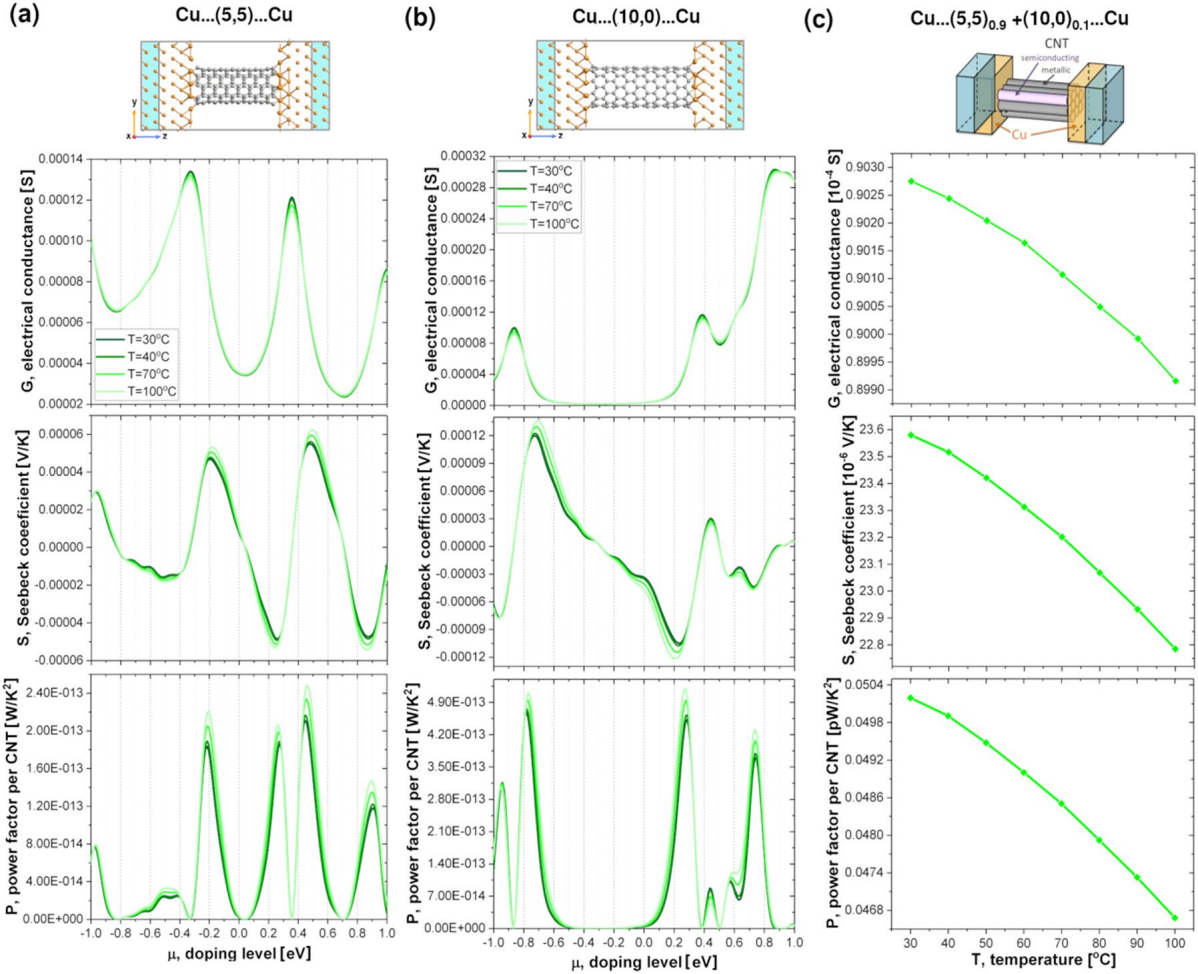
Computed thermoelectric properties of pristine (**a**) metallic (5,5) and (**b**) semiconducting (10,0) CNTs and (**c**) mixed parallel CNT circuits sandwiched between Cu electrodes. (**a**,**b**) Conductance (G), Seebeck coefficient (S) and Power Factor per CNT (P) are plotted as a function of doping level (μ) for four different temperatures: 30, 40, 70, and 100 °C. The atomistic side view of the models used for the transport calculations are presented at the top of the figure. C atoms are depicted in grey while Cu atoms are shown in orange. The semi-infinite electrodes consisting of perfect copper are depicted in blue. (**c**) G, S, P at different temperatures for a pristine CNT film containing 90% of metallic (5,5) and 10% semiconducting (10,0) CNTs with μ = − 0.99 eV computed using CNT bundles model adopted from Hayashi et al.^65^. The 3D visualisation of the model is shown above.

To unravel the impact of the dopants on the intrinsic component of resistance, we performed first principle calculations on various pristine and halide-doped CNT systems. We start the presentation of our results with the thermoelectric properties of pristine CNTs. Due to computational costs and well known dependence of thermoelectric properties of CNTs on their diameters^58^ we have chosen (5,5) and (10,0) CNTs as models of metallic and semiconducting CNTs, respectively. The electron transport calculations have been performed mostly on the device models shown atomistically in Fig. 9a,b. As in experiments, pristine nanotubes were sandwiched between two semi-infinite copper electrodes. Cu belongs to a group of metals that interact rather weakly with the CNT^59–61^, and so some intrinsic electronic structure properties of CNTs are preserved when contact is made between Cu and CNT. The charge transfer that occurs at the interface between Cu and the CNT produces band bending, which enables the CNT’s valence band edge to align with the Fermi level of the Cu electrode^62^. Unfortunately, due to the coupling between Cu and CNTs and resulting reduced sp^2^ hybridization of CNT, the conductance of metallic CNT is decreased with the respect to the intrinsic conductance of pure CNTs^60,61^. On the other hand, Cu electrodes introduce the so-called metal-induced gap states^63,64^ around the Fermi level in an energy region where the density of states of the pure semiconducting CNT does not have any non-zero values. The thermoelectric properties of CNTs coupled to CNT and Cu electrodes are compared in Fig. S6. In general, the thermoelectric properties of CNTs coupled to metal electrodes are more complicated functions of temperature (T) and chemical potential (μ) when coupled to the electrodes made of the same CNT.

Figure 9a,b show the electrical conductance, Seebeck coefficient and Power Factor per CNT as a function of chemical potential for the systems containing metallic and semiconducting single-walled CNTs, respectively. Due to the difficulty of defining the cross-sectional area of a CNT in a functional device we decided to calculate only electrical conductance (G) instead of conductivity (σ) to obtain the Power Factor per nanotube (P)^65^ rather than the absolute Power Factor (PF) defined in the introduction. The Power Factor per nanotube was calculated as S^2^G.

Due to pretreatment, the pristine CNT systems are already p-doped^16,66^, and the level of p-doping may vary depending on the experimental procedure. Therefore, it is important to analyse the thermoelectric properties of CNTs for a range of different chemical potentials^65,67^. As shown in the plots in Fig. 9a,b, it is possible to obtain either an increase or decrease of G, S and P with increasing T, for both types of tubes depending on their chemical potential. However, when metallic CNTs are coupled to the matching CNT electrodes, P only increases with increasing T for μ in the range of [− 1.4, 1.4] eV (see Fig. S6) Clearly analysis of thermoelectric properties of such a system, in contrast to the CNT coupled to Cu electrodes, does not reproduce experimental trends for CNT film that contains 90% of metallic nanotubes.

As previous works indicate^16,65,67^, the measured values of thermoelectric properties also depend strongly on the composition of the CNT system. Changing the ratio between semiconducting and metallic nanotubes can radically change the observed values of electrical conductivities, Seebeck coefficients and Power Factors. It might be necessary to take into account that 10% of CNTs in the film were semiconducting. To do so, we used simple model of mixed parallel CNT circuits introduced by Hayashi et al.^65^. Surprisingly, we were able to qualitatively reproduce our experimental trends (Fig. 9c). By using CNTs coupled to Cu electrodes and a chemical potential of − 0.99 eV, we obtained a decrease in all thermoelectric properties with increasing temperature. The conductance of the CNT film was calculated as:

$$G_{film} = 0.9G_{m} + 0.1G_{s} ,$$

while the Seebeck coefficient is calculated as:

$$S_{film} = \frac{{0.9G_{m} S_{m} + 0.1G_{s} S_{s} }}{{0.9G_{m} + 0.1G_{s} }},$$

where G_m(s)_ and S_m(s)_ are the conductances and Seebeck coefficients of systems presented in Fig. 9a,b, respectively. Even though this model neglects CNTs-CNTs junctions^67^, inelastic electron scattering and electron localization effects^65^, the computed electrical conductances are only three times higher than the experimental ones, while the Seebeck coefficients are only a factor of 2 smaller than the experimental values.

**Figure 10:**
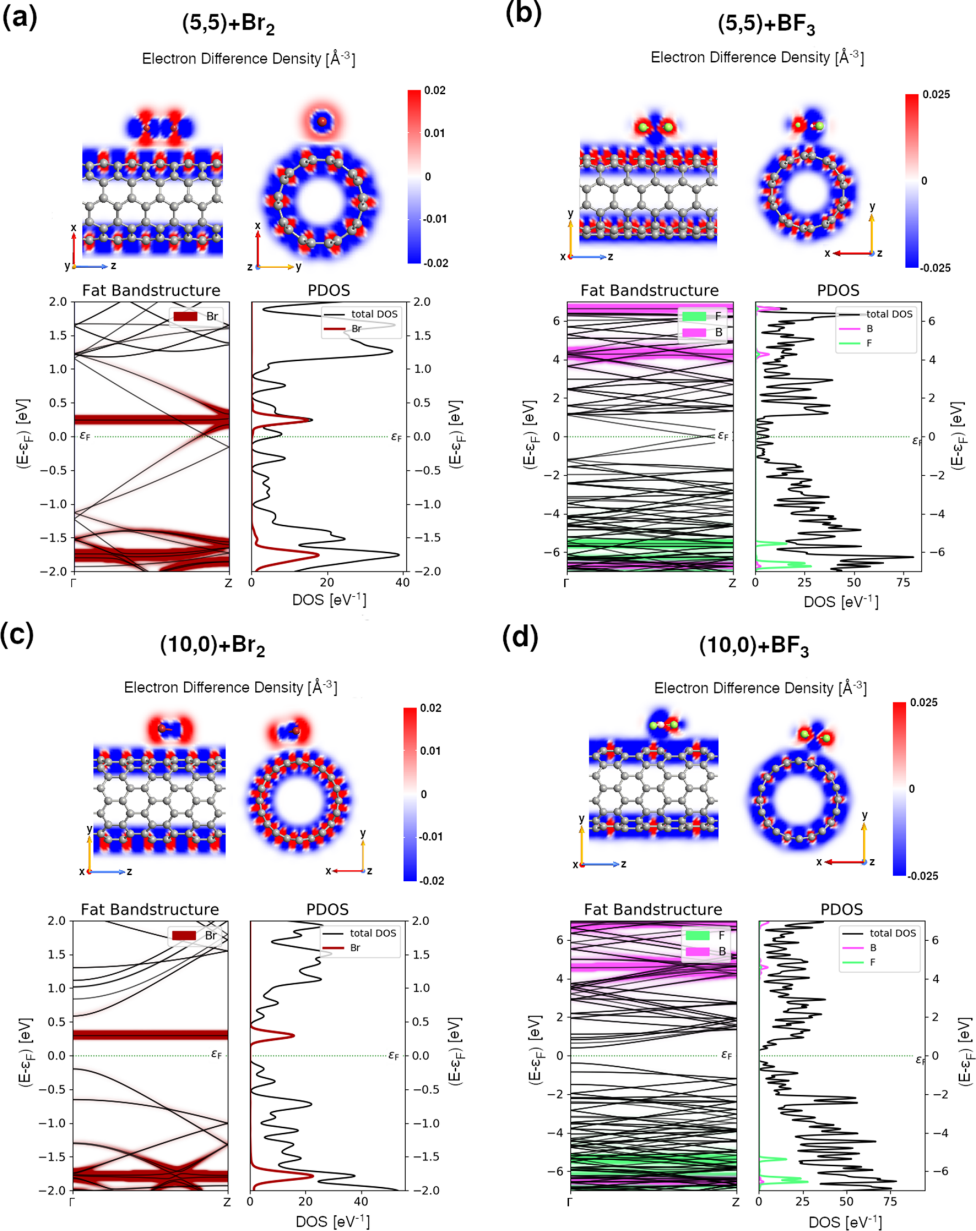
Computed electronic properties of (5,5) and (10,0) CNTs interacting with (**a**,**c**) Br_2_ and (**b**,**d**) BF_3_ molecules, respectively. (top panels) The electron different densities and (bottom left panels) fat band structures along Γ → Z of the Brillouin zone together with the projected density of states on chosen atomic species (PDOS) (bottom right panels). Br atoms are depicted in red while B, F and C atoms are shown in pink, light green, and grey, respectively.

Having described the pristine systems, we can now focus on the effect of halide doping. The influence of halide doping on CNTs was studied using (5,5), (10,0), (12,12) and (20,0) CNTs doped with Br_2_ and BF_3_ molecules, as shown in Fig. 10 and Fig. S7. The doping concentrations of small metallic (semiconducting) CNT correspond to 5.3 (4.5) and 11.7 (10.0) wt % of BF_3_ and Br_2_ molecules. When larger tubes are used, the concentrations of dopant reduce to 1.7 (1.7) and 3.8 (4.0) wt % of BF_3_ and Br_2_ molecules, respectively, which better corresponds to the experimental conditions. Even though the halide molecules create non-covalent bonding with CNTs, the interactions between them and the CNTs induce some structural changes that can be observed in all components of the system. As in previous works^21,66^, we also see small elongations of the bonds in both halide molecules compared to the isolated cases (see Tables S4 and S5). However, in agreement with experimental Raman spectroscopy results of this study, our calculations show that doping with both types of halide molecules does not significantly disturb CNT structures. The coefficient of radius variation (CV), which is a standardized measure of the change in the nanotube shape^68^, varies between only 0.0044 and 0.0008 for smaller tubes and between 0.0041 and 0.0004 for bigger tubes (see Tables S5 and S6). These values, in agreement with previous works on halide-doped CNT systems^21,66^, are from one to two orders of magnitude smaller than the ones observed after covalent functionalization of single-walled CNTs^68^. As expected, the CV values are higher for metallic tubes than for semiconducting CNTs. The tube deformation is also slightly higher after doping with BF_3_ molecules than with Br_2_ molecules. This is because BF_3_ molecules are located much closer to the CNTs than Br_2_ molecules. Although less negative adsorption energies per carbon atom (E_ads/NC_) of BF_3_-CNT systems than of Br_2_-CNT systems indicate that it is Br_2_ which creates stronger bond to CNTs.

The differences in the interaction between both dopant and CNTs are also clearly visible in the electron difference density (EDD) maps (see Fig. 10) which display the difference between the self-consistent valence charge density and the superposition of atomic valence densities. The blue regions indicate deficiency of electrons while red regions excess of electrons. Comparison between EDD maps of CNTs doped by Br_2_ and BF_3_ molecules suggests higher hole transfer from Br_2_ molecule to the nanotube than from BF_3_ to the nanotube. Note that these trends do not depend on the size of the nanotube (cf. Top panels in Fig. 10a–d and Fig. S7a–d).

Further analysis of electronic properties of doped CNTs (Tables S5 and S6) confirmed our experimental findings about the strength of p-doping induced by CNT functionalization with Br_2_ and BF_3_ molecules. Both functionalization routes produce p-type dopings which shift the Fermi level downwards. Doping with Br_2_ has much more profound impact on CNTs than BF_3_, especially when smaller tubes are used (cf. the Fermi level shift with respect to VBT for doping with Br_2_ and BF_3_).

In addition, electronic structure analysis (see Fig. 10 and Fig. S7) shows that additional states introduced by Br_2_ lie much closer to the Fermi level than these originating from the doping by BF_3_ (note the different scale on band structures and PDOS in Fig. 10 for systems containing Br_2_ and BF_3_). As in case of I_2_^21^, the flat impurity band originating from Br lowers the band gap of semiconducting tubes. The effect is particularly pronounced in small tubes (cf. E_gap_ values for pristine and doped (10,0) and (20,0) given in Tables S4 and S5). The original band gap of the (10,0) CNT was reduced by almost half after doping by Br_2_ (from 0.786 to 0.490 eV). As shown in Tables S5 and S6, the band gap reduction can be also observed when semiconducting tubes are doped with BF_3_ molecules. Note, however, that no additional impurity band is introduced in the gap region. Interestingly, adding BF_3_ to metallic tubes has the opposite effect, as it can create band gaps. Although, the resulting band gaps are small: 0.004 and 0.002 eV for (5,5) and (12,12) CNTs doped by BF_3_. These results explain why doping with Br_2_ gives higher increase in the electrical conductance of CNT systems than doping with BF_3._

**Figure 11:**
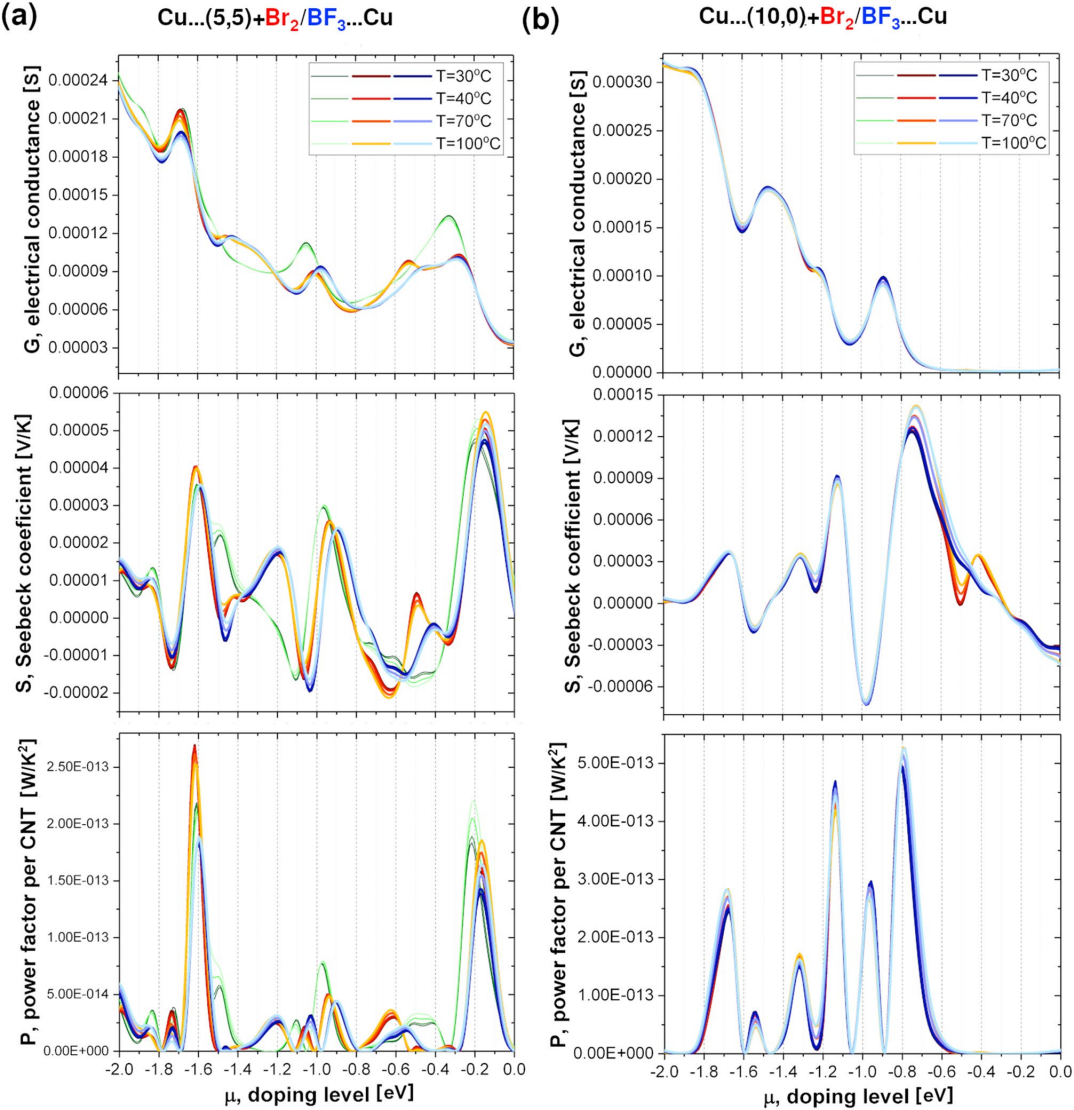
Computed thermoelectric properties of Br_2_ (red–orange lines) and BF_3_ (blue lines) doped (**a**) metallic (5,5) and (**b**) semiconducting (10,0) CNTs sandwiched between Cu electrodes. Conductance (G), Seebeck coefficient (S) and Power Factor per CNT (P) are plotted as a function of doping level (μ) for four different temperatures: 30, 40, 70, and 100 °C. Thermoelectric properties of pristine CNTs (green lines) are shown as a reference.

To further understand the impact of halide doping on CNT systems we have performed transport calculations for metallic and semiconducting CNTs doped with Br_2_ and BF_3_ molecules. As in case of pristine CNTs, doped CNTs were sandwiched between two semi-infinite copper electrodes. The thermoelectric properties of doped CNTs with respect to the pristine CNTs are presented in Fig. 11. For convenience, only the p-doping region (negative values of chemical potential) is shown. The differences between pristine, Br_2_- and BF_3_-doped systems for each thermoelectric property are clearly visible for metallic CNT (Fig. 11a), while G, S or P looks similar regardless of the presence of doping and its type for semiconducting CNT (Fig. 11b).

This indicates the importance of the initial treatment of CNT film that is mainly composed of metallic tubes. According to calculations, the observed trend in G, S, P for halide-doped CNTs is non-obvious. It is possible to obtain either a higher or lower G, S and P of pristine, Br_2_- and BF_3_-doped CNTs for a given temperature, depending on the chemical potential. Moreover, as in the case of pristine tubes, the temperature dependence of G, S, P of halide-doped CNTs is also determined by the value of μ. Therefore, it is unambiguously one of the key parameters dictating the electronic behavior of the network. The results of the computations using the model of mixed parallel CNT circuits do not fully reproduce the experimental trends shown in Figs. 4, 5 and 6. The investigated films are composed of numerous types of CNTs, which very much complicates the transport properties calculations. As highlighted by Li and co-workers^69^, unsorted material of this diameter range (1.8 ± 0.4 nm) may contain more than a hundred chiralities. Therefore, it cannot be excluded, that there is some chirality influence on the extent how much a halide compound interacts with a given CNT type. This would explain why simple modelling of the system by only two types of halide-doped CNTs was not fully successful.

It must also be noted that the concentration of dopants in the systems used for the calculations were an order of magnitude higher than in the experimental samples. It would be challenging to conduct computation mimicking the selected experimental conditions as CNTs of large diameter would need to be extended considerably to adjust the concentration of the halide compounds. Both of these factors may explain the aforementioned discrepancy. Nevertheless, this disparity does not invalidate the main finding of the manuscript that the chemical potential of the CNTs is an essential factor to consider when investigating doping of CNT ensembles.

## Conclusions

CNTs doped with compounds containing a halide atom show significantly enhanced electrical conductivity, which is due to interactions at the surface between the electronegative atoms and the band structure of the CNTs. As for the materials we tested, as the sample temperature increased, the electrical conductivity decreased, which shows that the CNT networks are of predominantly metallic character.

For the pristine CNT film, it was observed that the rise in temperature decreases the value of the Seebeck coefficient. On the other hand, an increase in this property was observed for all the doped materials. This behavior had a direct impact on the Power Factor value. At room temperature, only two materials (doped with boron trifluoride and 2,4,5-trichlorophenol) showed a Power Factor higher than the neat CNT material. However, as the temperature increased, all the doped CNT films revealed notable enhancements in PF at temperatures of 100 °C. This is an important finding because thermoelectric generators are envisioned to operate at elevated temperatures. Yet, most commonly, the nanocarbon capabilities in this field are reported in the literature for conditions close to ambient ones.

Our calculations showed that thermoelectric properties of CNT systems are determined by the chemical potential of the starting CNT films. The electrical conductivity, Seebeck coefficient and Power Factor of doped CNT systems strongly depend on the metallic to semiconducting CNT ratio, the level of initial doping and temperature. These factors are particularly important for systems predominantly composed of metallic CNTs.

Finally, thermogravimetric tests showed that most of the doped materials studied are stable at least up to 100 °C. Such an improvement in Power Factor value and the verified thermal stability suggest that materials of this type can be used in thermogenerators receiving thermal energy generated e.g. by car engines, where temperatures near the engine block of ca. 95 °C are readily accessible. We believe that the advantages of CNTs compared to the materials based on rare earth elements can potentially make a significant contribution in this field once key problems are overcome (chirality control, alignment, and contact resistance). With regard to doping agents, further research is required to fully understand how their structure and chemical nature affect the thermoelectric performance of nanocarbon to obtain the highest possible enhancement and stability; however, the present work provides a clear basis for such further studies.

## Methods

### Materials

In our study, we used high-quality single-walled CNTs (SWCNTs) purchased from OCSiAl, the brand name of which is Tuball (lot: O1RW02.N1.382; date of production: 10 May 2018).

Ethyl cellulose (EC; ethoxyl content 48%), purchased from Acros Organics, was used as the binding agent during the process of thin film production.

Bromine, iodine monochloride, boron trifluoride, titanium (III) chloride, tribromomethane, 1,2-dibromoethane, 1,1,2,2-tetrachloroethane, 1,1,1-trifluoroacetone, 2-chloroacetic acid, trifluoroacetic acid, trifluoroacetic anhydride, ethyl chloroacetate, chloroacetonitrile, epibromohydrin, hexachlorocyclopentadiene, chlorobenzene, m-dichlorobenzene, p-dichlorobenzene, p-bromotoluene, 2,4-dichlorophenol, 2,4,5-trichlorophenol, p-chlorobenzaldehyde, 4-chloroacetophenone, m-chlorobenzoic acid, tetrachlorophthalic anhydride, 2-chloropyridine, and 1-(2-ethoxy-2-oxoethyl)pyridinium bromide were used as doping agents. All the reagents have pure p.a. class and were purchased from Acros Organics, Merck, Sigma Aldrich, or Fluka. Bromine and boron trifluoride were in the form of aqueous and methanol solutions, respectively.

Toluene and acetone were used as the dispersion environment in the production of CNT films. Methanol, dichloromethane, acetone, and distilled water were used to create the doping solutions. All the solvents (apart from distilled water obtained in-house) were pure p.a. and purchased from ChemPur, Poland. Furthermore, the solvents were all verified to ensure that they themselves do not cause changes in the electrical or thermoelectric properties. No differences were discerned; Fig. S4 shows an example of Seebeck coefficient measurement result obtained for a pristine CNT film with and without dipping it in acetone after drying.

### Preparation of CNT films

In order to measure the electrical and thermoelectric properties of the CNTs doped with halide compounds, it was necessary to obtain thin, free-standing films. The method used for this purpose was described in our earlier publications^20,70^. In short, it consists of creating a 0.55 g SWCNT dispersion with ethyl cellulose (1:10 weight ratio) within 66.5 g of an ice-cold mixture of toluene and acetone (1:1). After obtaining a homogenous mixture by sonication (Hielscher, UP200St, 100% amplitude, 15 min.), the CNT paint was deposited onto a Kapton foil, to which the resulting CNT film had low adhesion. Once the entire solvent evaporated from the film, it was slowly peeled off from the substrate. In the final step, the binder (ethyl cellulose) was removed by annealing in air, which took no more than a couple of seconds while keeping the properties of the material intact.

### Doping of CNT films

Doping was carried out using the dipping method. Strips of 2 × 50 mm were cut out of the CNT films. They were immersed in 0.1 M solution of the dopant for 30 s. An overview of the compounds used in the experiment is presented in Table S3. After immersion, CNT films were dried in a vacuum desiccator. Depending on the doping substance, the solvent used was water, acetone, methanol, or dichloromethane (DCM). In this study, it was necessary to use different solvents due to the different chemical nature of the dopants.

### Characterization

The Raman spectra (Renishaw, λ = 514 nm laser, 10% power) were collected using the extended recording mode from 0 to 3500 cm^−1^ and the integration time of 10 s to increase the signal to noise ratio. For every sample, the measurement was repeated in at least three sample locations using 3 accumulations. The Raman spectra were used to determine the influence of the doping agent on the electronic characteristics of CNT films and to verify whether their addition causes chemical modification of the material.

The thermal stability of the CNTs doped with halide compounds was characterized by a thermogravimetric analyzer (Mettler Toledo TGA/DSC 1 STAR) in the temperature range from 25 to 1000 °C in a flow of air (30 mL/min) and with the heating rate of 10 °C/min.

The changes in electrical conductivity of the samples after doping with halide compounds were measured using the 4-point method. The tests were conducted using the Keithley 2450 SourceMeter, and the electrical resistivity was measured under 100 mA electric current. The absolute value of electrical conductivity was calculated based on the dimensions of samples measured with a 4″ digital caliper (Hi-Tech diamond) for width and length, as well as a Multi-Anvil Micrometer (Electronic Universal IP54, Linear) for thickness. The electrical conductivity of all the samples was initially measured at room temperature. For the most promising dopants, measurements were also taken at higher temperatures (40 °C, 70 °C, and 100 °C) by heating the samples on a hot plate while recording the sample temperatures in a non-contact mode by Infrared Thermometer Scan Temp 380 (TFA).

The Seebeck coefficient was determined with the use of a custom-made setup (SeebCam 2018, LBR, Lublin, Poland). It was measured in the range of temperatures from 30 to 100 °C for the samples of the size of 2 × 50 mm. The sample was placed on a board maintained in a sealed chamber to eliminate the convection effect (Fig. S8). Both ends of the sample had appropriate contact with temperature sensors and resistive heaters. To ensure a suitable electrical and thermal interface between the sample and the equipment, silver paint SCP Electrolube was used. The electric potential difference between the sample ends was measured (Keithley 2128A) at the temperature gradient of 5 °C in the temperature range between 30 °C and 100 °C. The temperatures mentioned in the text refer to the temperatures of the hot end. Each sample was subject to at least 5 measurements. The results were averaged, and the statistical error was calculated.

The Power Factor value was calculated from the measured values of electrical conductivity and Seebeck coefficient.

Scanning Electron Microscope (SEM, FEI Quanta 250 FEG) running at the acceleration voltage of 15 kV was used to study the microstructure of the selected materials. The samples were not sputtered with metal because of their high electrical conductivity.

X-ray photoelectron spectroscopy (XPS) measurements were performed in an ultra-high vacuum system (base pressure ca. 8 × 10^–9^ Pa) with a PREVAC EA-15 hemispherical electron energy analyzer equipped with the 2D-MCP detector. In order to provide the best possible energy resolution, the Mg Kα energy line (1253.60 eV; PREVAC XR-40B dual-anode source) was used as well as the curved analyzer slit was applied. The pass energy was set to 200 eV for the survey spectra, with a step of 0.9 eV and set to 100 eV for individual energy regions, with an energy step of 0.05 eV. The binding energy (BE) scale of the analyzer was calibrated to Ag 3d 5_/2_ (368.2 eV)^71^. The energy region decomposition was performed with the use of CASA XPS software. Each peak was represented by a sum of Gaussian (70%) and Lorentzian (30%) lines. For the background subtraction, the Shirley function was utilized. The full width at half maximum (FWHM) of the components at the same energy region were allowed to vary within a narrow range.

Optical absorption spectroscopy was used to estimate the ratio of metallic to semiconducting CNTs. CNTs (1 mg/mL) in 1%wt aqueous sodium cholate solution were homogenized by tip-sonication (1 h, Hielscher UP200St), and then ultracentrifuged (1 h, 15,314×*g*, Eppendorf Centrifuge 5804 R) to remove the non-individualized fraction. The supernatant was investigated in the wavelength range of 400–1100 nm using the Hitachi U-2910 spectrophotometer.

### Modelling

The structural and electronic properties of pristine and doped CNT systems have been carried out in the framework of the spin-polarized density functional theory (DFT)^72,73^ in the generalised gradient approximation (GGA) approach using Perdew-Burke-Ernzerhof (PBE) parameterization^74^ as implemented in QuantumATK^75,76^ and Siesta^77,78^ numerical packages. Valence electrons were represented with double-ζ numerical basis (DZP) sets of orbitals localized on atoms, with polarization functions also included. The influence of the core electrons was accounted for using norm-conserving Troullier–Martins^79^ nonlocal pseudopotentials cast in the Kleinman–Bylander^80^ separable form. Long-range interactions between the halides and the CNTs were included in the total bonding energy using the methodology proposed by Grimme^81^. The Brillouin zone was sampled in 3 × 3 × 7 Monkhorst and Pack scheme^82^, while the density mesh cutoff for real-space integrals was set to 300 Ry. For the band structure and density of states calculations, the kinetic cut-off for real-space integrals was increased to 500 Ry. During all calculations, the self-consistent field (SCF) cycle was iterated until the density matrix by less than 10^−5^ per iteration. Models presented in Fig. S9 were optimized until a maximum force converged to lower than 0.004 eV/Å and the total energy changed by less than 10^−5^ eV. The simulations were performed using 3D periodic boundary conditions.

The electronic coherent transport has been studied employing the non-equilibrium Green’s function method (NEGF) as implemented in QuantumATK^75,83^. The computed structures were treated as two-probe systems with the central scattering region coupled to the fully relaxed semi-infinite electrodes as shown in Fig. 9a,b. Both interfaces between the Cu (100) surfaces and the CNT open ends were fully relaxed. The Brillouin zone of the two-probe system was sampled using a 3 × 3 × 101 Monkhorst–Pack scheme. The transmission spectra were calculated using increased 7 × 7 sampling in the [− 3,3] eV range within 601 points. The basis sets were reduced to SZP, while other parameters remain the same as in the previous paragraph.

The electrical current through the device under a finite bias voltage, U, can be defied according to Landauer-Büttiker formula^84^ as: $I(U) = \frac{2e}{h}\int_{ - \infty }^{\infty } {T(\varepsilon ,\;U)\left( {f_{L}^{FD} \left( {\frac{{\varepsilon - \mu_{L} }}{{K_{B} T_{L} }}} \right) - f_{R}^{FD} \left( {\frac{{\varepsilon - \mu_{R} }}{{K_{B} T_{R} }}} \right)} \right)d\varepsilon ,}$ where T(ε, U) is the energy and voltage-resolved transmission function, $\mu_{L} (R) = \varepsilon_{F} \pm eU/2$ represent the electrochemical potential of the left (right) electrode, $f_{L(R)}^{FD}$ and T_L(R)_ are the corresponding Fermi–Dirac electron distribution and temperature. ε_F_ is the Fermi energy. The transmission function of electrons with energy ε incident in the central scattering region constituting the device can be expressed using the retarded Green’s function $\hat{G}(\varepsilon )$: $T(\varepsilon ) = Tr\left[ {\hat{G}(\varepsilon )\widehat{{\Gamma_{L} }}(\varepsilon )\hat{G}(\varepsilon )^{\dag } \widehat{{\Gamma_{R} }}(\varepsilon )} \right],$ where $\hat{\Gamma }_{L(R)} (\varepsilon ) = i\left( {\hat{\Sigma }_{L(R)} (\varepsilon ) - \hat{\Sigma }_{L(R)} (\varepsilon )^{\dag } } \right)/2,$ is the broadening function of left (right) electrode, and $\hat{\Sigma }_{L(R)} (\varepsilon )$ are the corresponding self-energies. The thermoelectric coefficients were calculated using the linear response approximation, where the electrical conductance and the Seebeck coefficient are defined as^58,61,74,76^: $G = \left. {\frac{dI}{{dU}}} \right|_{dT = 0} = \frac{1}{U} \times \Delta \mu \frac{2e}{h}\int_{ - \infty }^{ - \infty } {T(\varepsilon )\frac{\partial f(\varepsilon ,\mu ,T)}{{\partial \mu }}d\varepsilon }$ and $S = \left. { - \frac{dU}{{dT}}} \right|_{I = 0} = - \frac{1}{e} \times \frac{{\frac{2e}{h}\mathop \smallint \nolimits_{ - \infty }^{ - \infty } T(\varepsilon )\frac{\partial f(\varepsilon ,\mu ,T)}{{\partial T}}d\varepsilon }}{{\frac{2e}{h}\mathop \smallint \nolimits_{ - \infty }^{ - \infty } T(\varepsilon )\frac{\partial f(\varepsilon ,\mu ,T)}{{\partial \mu }}d\varepsilon }}$, respectively.

Computed parameters for (5,5) and (10,0) as well as (12,12), (20,0) CNTs are given in Table S4 and S5, respectively.

## Supplementary Information

Supplementary Information.
