# Supplementary material for: Enhancing thermoelectric properties of single-walled carbon nanotubes using halide compounds at room temperature and above
Source: Sci Rep. 2021 Apr 21;11:8649. doi: 10.1038/s41598-021-88079-w (PMC8060344; doi:10.1038/s41598-021-88079-w)
Supplement: Supplementary file 1 — Supplementary Information. [file 41598_2021_88079_MOESM1_ESM.docx]

Supplementary Information

**Enhancing thermoelectric properties of single-walled carbon nanotubes using halide compounds at room temperature and above**

Bogumiła Kumanek^a^*, Grzegorz Stando^a^, Paweł Stando^a^, Karolina Matuszek^b^,
Karolina Z. Milowska^c^, Maciej Krzywiecki^d^, Marta Gryglas-Borysiewicz^e^,
Zuzanna Ogorzałek^e^, Mike C. Payne^c^, Douglas MacFarlane^b^, Dawid Janas^a^*

^a^ Department of Organic Chemistry, Bioorganic Chemistry and Biotechnology, Silesian University of Technology, B. Krzywoustego 4, 44-100 Gliwice, Poland

^b^ Monash University, School of Chemistry, Clayton, VIC 3800, Australia

^c^ TCM Group, Cavendish Laboratory, University of Cambridge, 19 JJ Thomson Avenue, Cambridge CB3 0HE, United Kingdom

^d^ Institute of Physics-CSE, Silesian University of Technology, Konarskiego 22B, 44-100, Gliwice, Poland

^e^ Faculty of Physics, University of Warsaw, Pasteura 5, 02-093 Warsaw, Poland

*Corresponding authors: [bogumila.kumanek@polsl.pl](mailto:bogumila.kumanek@polsl.pl); [dawid.janas@polsl.pl](mailto:dawid.janas@polsl.pl)

Table S1 Electrical and thermoelectric properties of CNTs doped with halide compounds

| Base material | Doping agent | Electrical conductivity (at 300K) [S/m] | Seebeck coefficient [μV/K] | Power Factor  [µW/m·K^2^] | Ref. |
| --- | --- | --- | --- | --- | --- |
| s-SWCNTs | pristine | 7.3∙10^4^ | - | - | [1] |
|  | HSO_3_Cl | 1.9∙10^5^ | - | - |  |
|  | IBr | 2.2∙10^5^ | - | - |  |
|  | I_2_ (vapour) | 2.4∙10^5^ | - | - |  |
|  | KAuBr_4_ | 4.3∙10^5^ | - | - |  |
| m-SWCNTs | pristine | 9.0∙10^4^ | - | - | [1] |
|  | HSO_3_Cl | 3.2∙10^5^ | - | - |  |
|  | IBr | 1.5∙10^5^ | - | - |  |
|  | I_2_ (vapour) | 1.0∙10^5^ | - | - |  |
|  | KAuBr_4_ | 2.4∙10^5^ | - | - |  |
| s-SWCNTs  (laser vaporization) | pristine | 408 Ω/□ | - | - | [2] |
|  | SOCl_2_ (treated by HNO_3_) | 136 Ω/□ | - | - |  |
| m-SWCNTs  (laser vaporization) | pristine | 212 Ω/□ | - | - | [2] |
|  | SOCl_2_ (treated by HNO_3_) | 124 Ω/□ | - | - |  |
| SWCNTs  (HiPco) | pristine | 0.5∙10^6^ | - | - | [3] |
|  | HSO_3_Cl | 3.0∙10^6^ | - | - |  |
|  | I_2_ | 5.5∙10^6^ | - | - |  |
| SWCNTs | pristine | 6.25∙10^4^ | -  - | -  - | [4] |
|  | Br_2_ (vapour) | 1.0∙10^6^ |  |  |  |
|  | pristine | 5.5∙10^4^ | - | - |  |
|  | SOCl_2_ | 3.0∙10^5^ | - | - |  |
| SWCNTs  (HiPco) | pristine | 5.5∙10^4^ | - | - | [5] |
|  | I_2_ | 1.45∙10^5^ | - | - |  |
|  | SO_2_Cl_2_ | 8.2∙10^4^ | - | - |  |
|  | PyPhF_5_ | 0.37∙10^4^ | - | - |  |
|  | PhCH_2_Cl | 4.0∙10^4^ | - | - |  |
|  | PhCH_2_Br | 1.3∙10^4^ | - | - |  |
| SWCNTs  (arc discharge) | pristine | 7.5∙10^4^ | 24 | 43.2 | [6] |
|  | HSO_3_Cl | 3.1∙10^5^ | 28 | 240.03 |  |
| SWCNTs  (HiPco) | pristine | 7.5∙10^3^ | 32 | 7.68 | [6] |
|  | HSO_3_Cl | 1.1∙10^5^ | 30 | 99 |  |
| SWCNTs (CVD) | pristine | 1.0∙10^4^ | 45 | 20.25 | [6] |
|  | HSO_3_Cl | 7.0∙10^4^ | 30 | 153 |  |
| SWCNTs (CVD) | pristine | 3.5∙10^3^ | 49 | 8 | [7] |
|  | (F-Ph)_3_P | 3∙10^3^ | -6.3 | 0.11 |  |
|  | (Cl-Ph)_3_P | 2.6∙10^3^ | -29 | 2.2 |  |
| DWCNTs (CVD) | pristine | 2.2∙10^5^ | 34 | 254.32 | [6] |
|  | HSO_3_Cl | 1.7∙10^6^ | 18 | 550.80 |  |
| DWCNTs (CVD) | pristine | 2.85∙10^4^ | - | - | [8] |
|  | I_2_ (vapour) | 5.9∙10^4^ | - | - |  |
| DWCNTs (CVD) | pristine | 1∙10^6^ | - | - | [9] |
|  | I_2_ (vapour) | 6.67∙10^6^ | - | - |  |
| Abbreviations: s – semiconducting, m – metallic, CVD – chemical vapour deposition, Py – pyridine group, Ph – phenyl group. | | | | | |


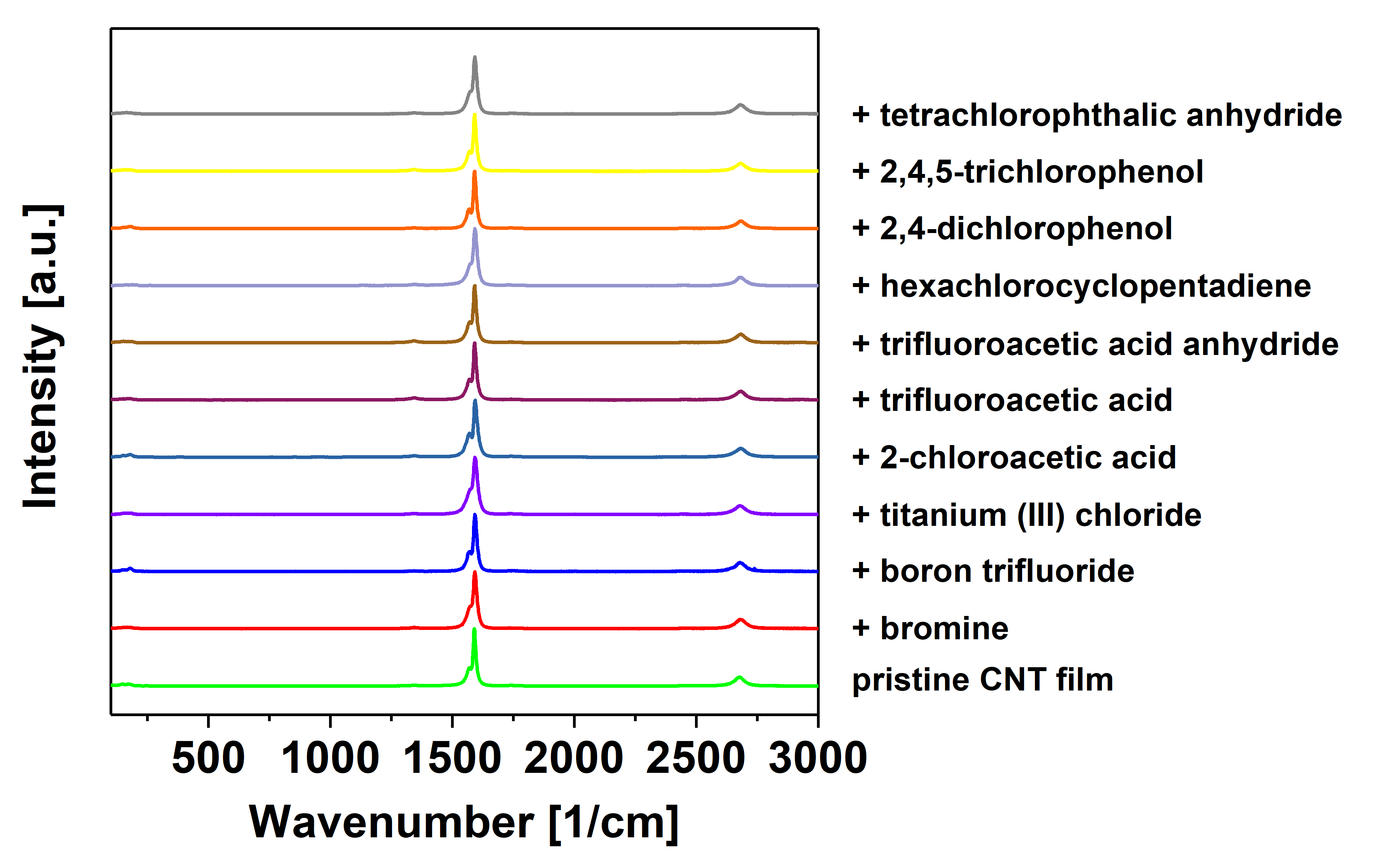


Figure S1 Raman spectra for the selected samples.

Table S2 shows comparison of results of TGA analysis. The temperature of the maximum weight loss was marked as T_max_. The remaining sudden weight loss signals were marked as T_loc_ (loc – local maxima). The last column states the boiling temperature (T_b_) or decomposition temperature (T_d_) for the corresponding substances taken from the literature as a comparison.

Table S2 The influence of doping on thermal stability of CNT films measured by TGA.

| No. | Sample | T_max_ [°C] | T_loc_  [°C] | T_b_ or T_d_  [°C] |
| --- | --- | --- | --- | --- |
|  | Pristine CNT film | 651 | 747 |  |
| 1 | + Bromine | 679 | 93  173  595 | 58.8 |
| 2 | + Boron trifluoride | 729 | 56  653 | - |
| 3 | + Titanium (III) chloride | 711 | 335  665 | 425 |
| 4 | + Hexachlorocyclopentadiene | 751 | 223  336 | 239 |
| 5 | + 2,4-dichlorophenol | 739 | 213  360 | 209 |
| 6 | + 2,4,5-trichlorophenol | 740 | 230  374  568 | 253 |
| 7 | + 2-chloroacetic acid | 744 | 65  185  349 | 189 |
| 8 | + Trifluoroacetic acid | 644 | 53  250  374  751 | 73.4 |
| 9 | + Trifluoroacetic anhydride | 642 | 48  261  364  712 | 40 |
| 10 | + 2,4,6,7-tetrachlorophthalic anhydride | 598 | 73  148  331  409  738 | 371 |


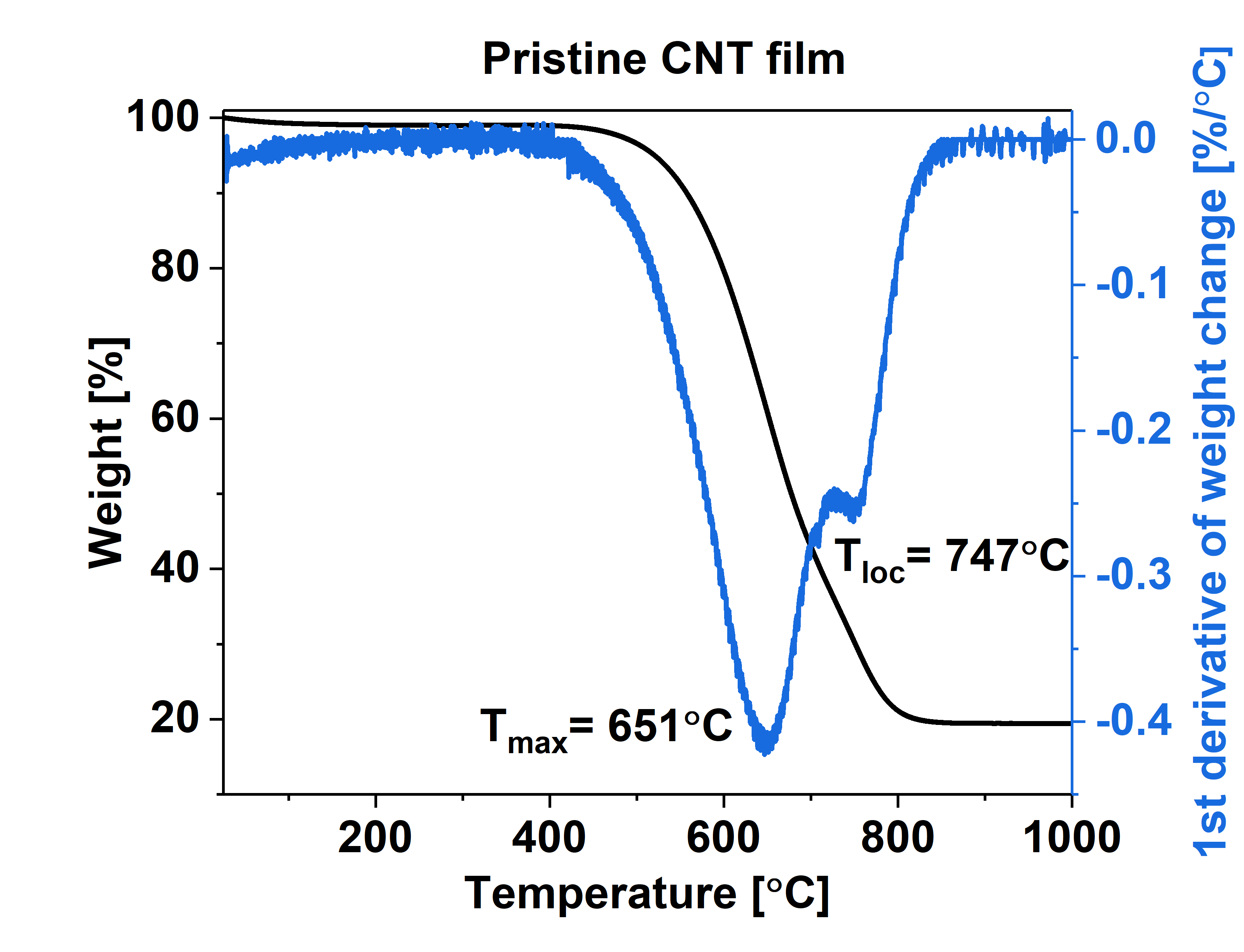


Figure S2 TGA for pristine CNT film with first derivative of weight change with respect to the temperature.


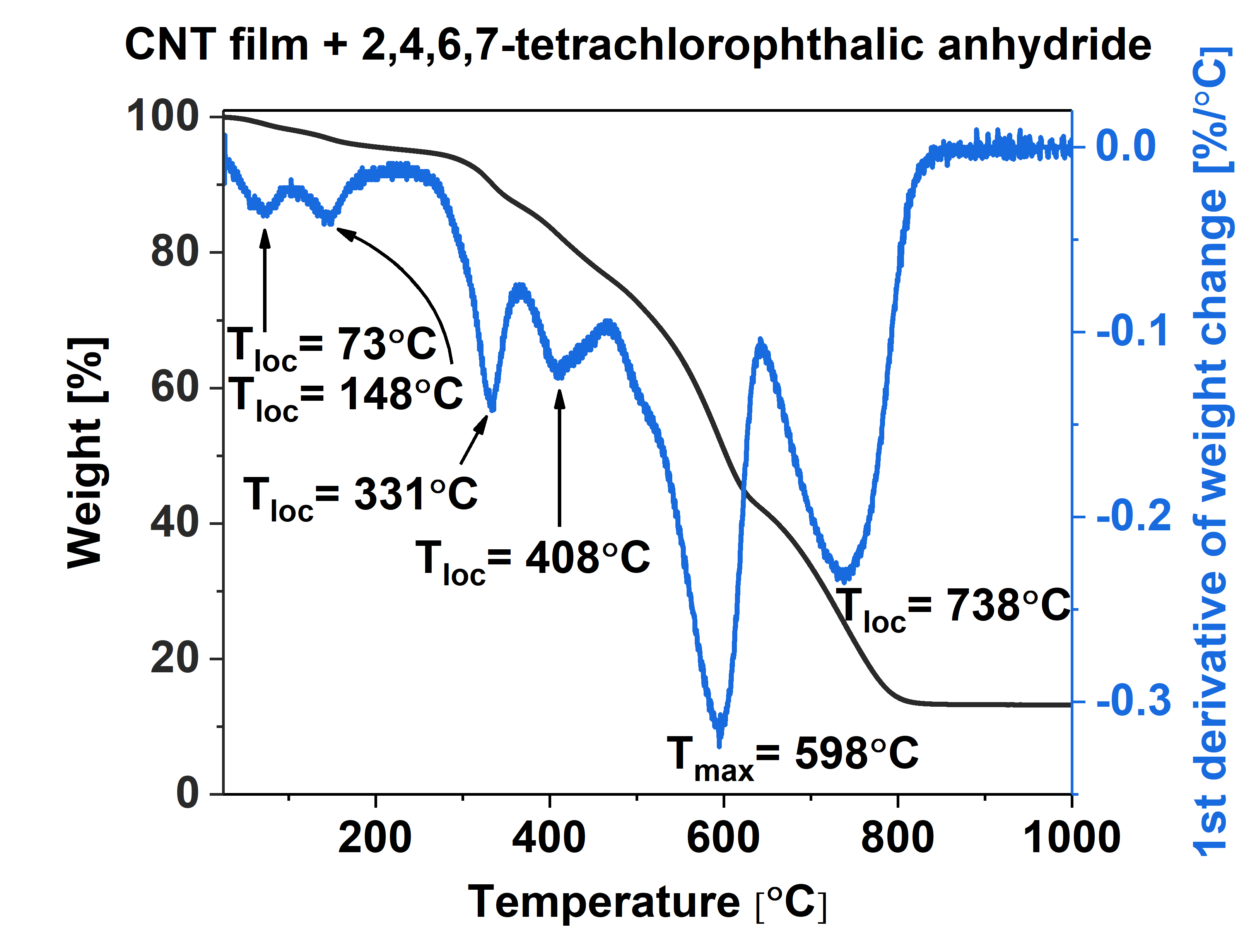


Figure S3 TGA for sample of CNT film doped with 2,4,5,7-tetrachlorophthalic anhydride with first derivative of weight change with respect to the temperature.


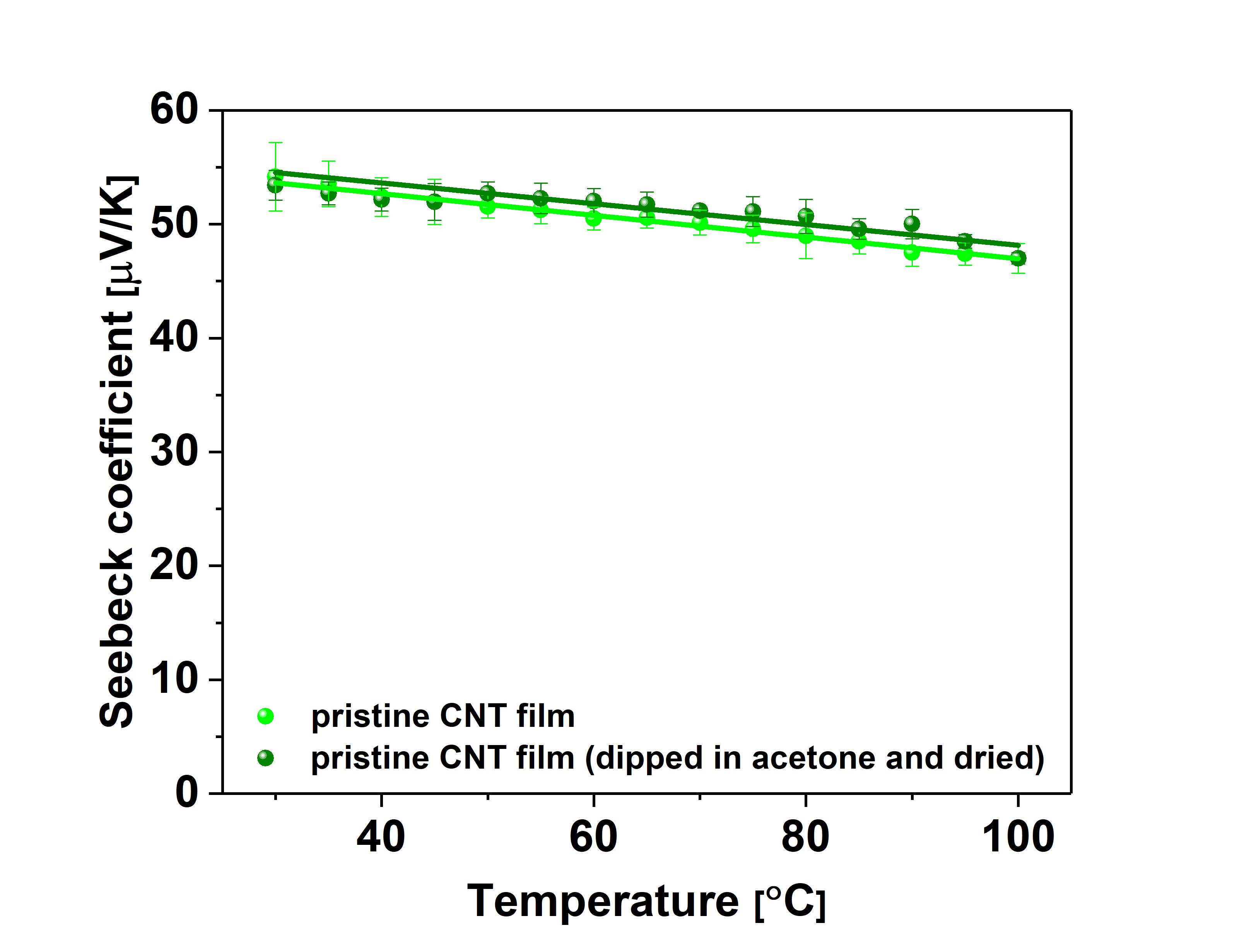


Figure S4 Seebeck coefficients of pristine CNT films before and after dipping in acetone and drying.


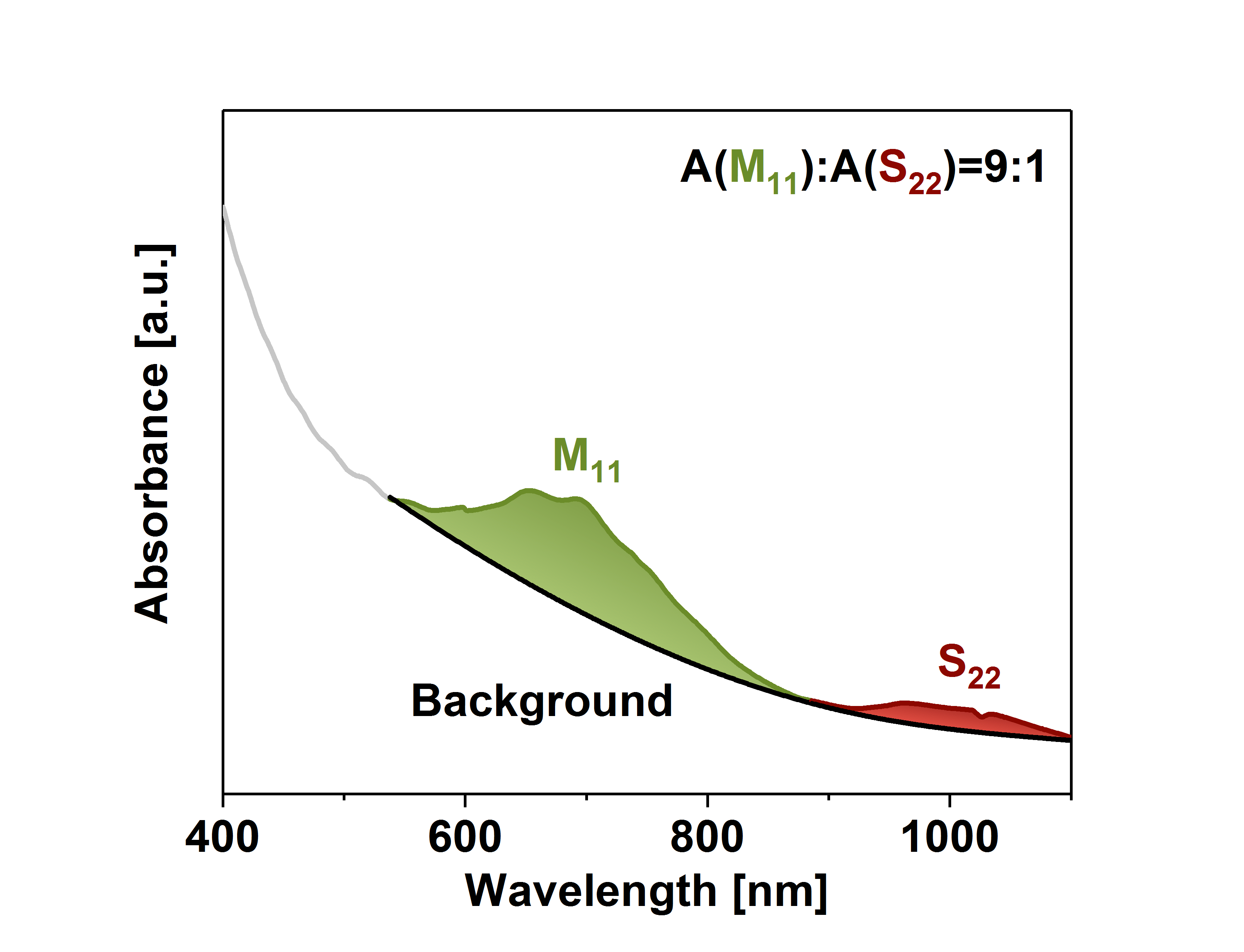


Figure S5 Estimation of the content of metallic and semiconducting fraction in the material by the optical absorption spectroscopy method.


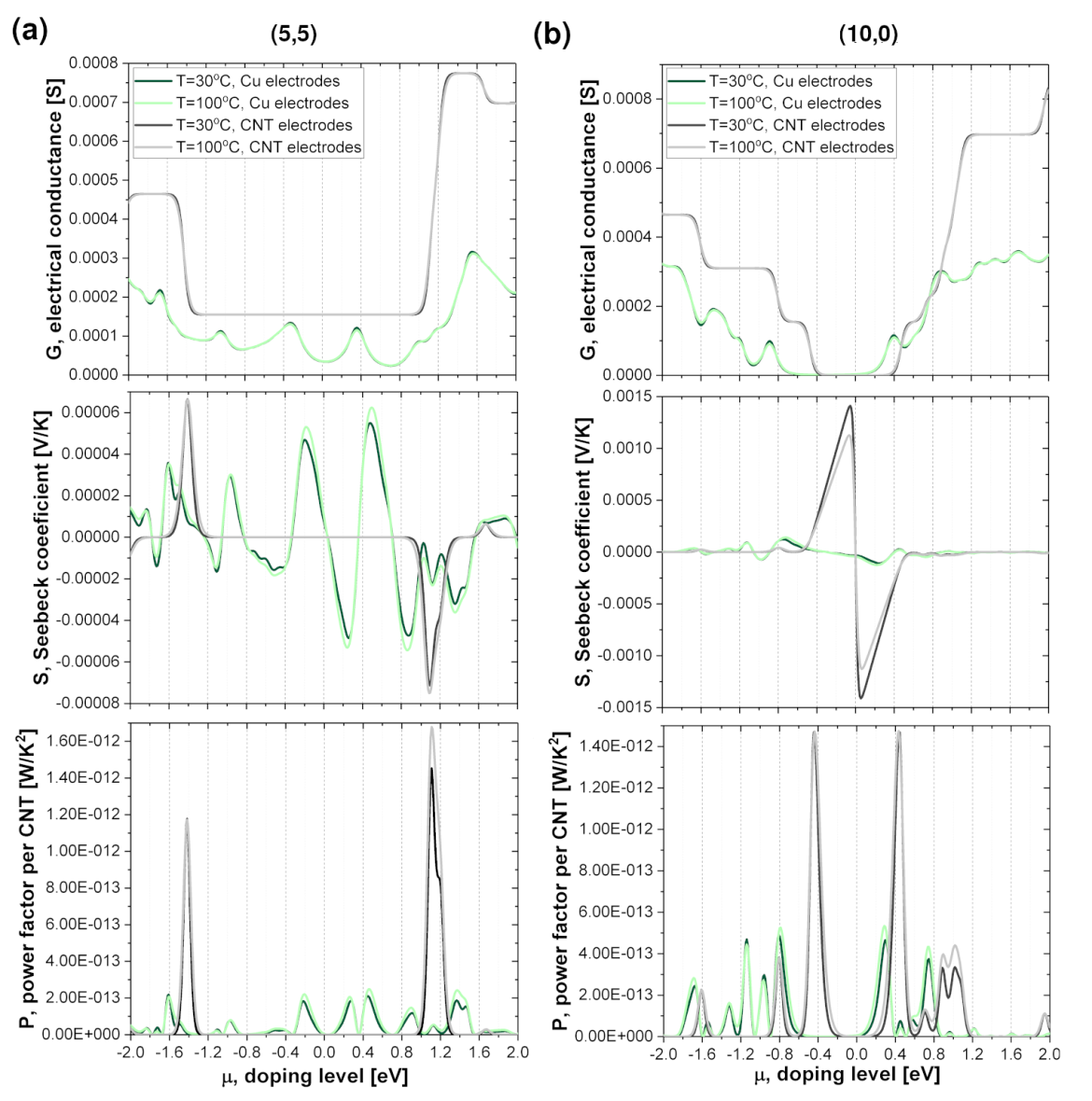


Figure S6 Computed thermoelectric properties of pristine (a) metallic (5,5) and (b) semiconducting (10,0) CNTs with semi-infinite CNT (grey lines) and Cu electrodes (green lines). Conductance (G), Seebeck coefficient (S) and power factor per CNT (P) are plotted as a function of doping level (μ) for two different temperatures: 30 and 100 ^o^C.


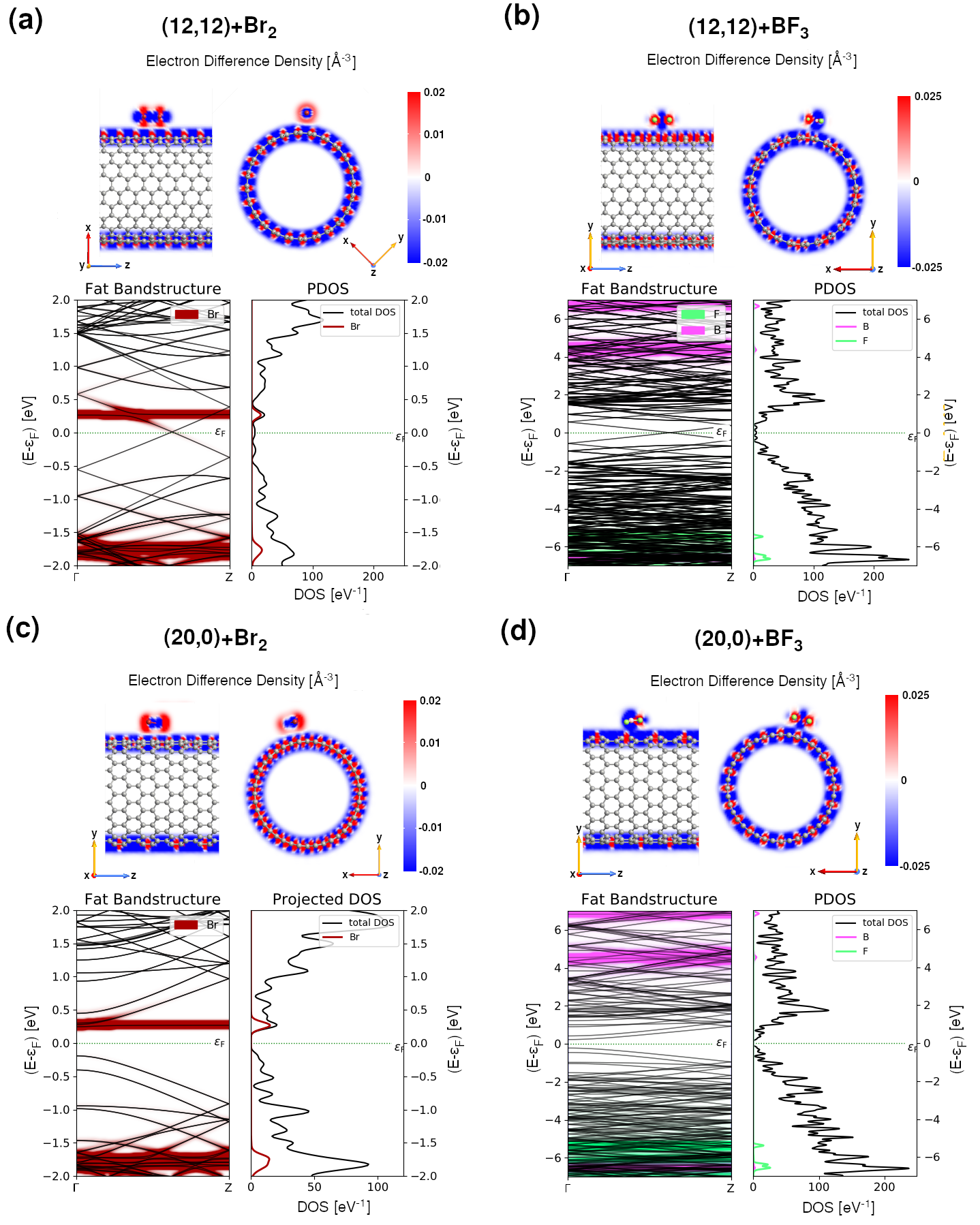


Figure S7 Computed electronic properties of (12,12) and (20,0) CNTs interacting with (a,c) Br_2_ and (b,d) BF_3_ molecules, respectively. (top panels) The electron different densities and (bottom left panels) fat band structures along Γ → Z of the Brillouin zone together with the projected density of states on chosen atomic species (PDOS) (bottom right panels). Br atoms are depicted in red while B, F and C atoms are shown in pink, light green, and grey, respectively.

Table S3 presents the compounds used for doping CNT films. In addition, the table also contains information about the solvent used, the 0.1M solution of which was made for the doping of the films.

Table S3 Information about doping agents used in the study.

| No. | Name | Solvent |
| --- | --- | --- |
| Inorganic compounds | | |
| 1 | Bromine | water |
| 2 | Iodine monochloride | DCM |
| 3 | Boron trifluoride | methanol |
| 4 | Titanium (III) chloride | acetone |
| Organic compounds | | |
| 5 | Tribromomethane | DCM |
| 6 | 1,2-dibromoethane | acetone |
| 7 | 1,1,2,2-tetrachloroethane | acetone |
| 8 | 1,1,1-trifluoroacetone | acetone |
| 9 | 2-chloroacetic acid | acetone |
| 10 | Trifluoroacetic acid | acetone |
| 11 | Trifluoroacetic anhydride | acetone |
| 12 | Ethyl chloroacetate | acetone |
| 13 | Chloroacetonitrile | DCM |
| 14 | Epibromohydrin | acetone |
| 15 | Hexachlorocyclopentadiene | DCM |
| 16 | Chlorobenzene | DCM |
| 17 | m-dichlorobenzene | DCM |
| 18 | p-dichlorobenzene | DCM |
| 19 | p-bromotoluene | DCM |
| 20 | 2,4-dichlorophenol | acetone |
| 21 | 2,4,5-trichlorophenol | acetone |
| 22 | p-chlorobenzaldehyde | acetone |
| 23 | 4-chloroacetophenone | DCM |
| 24 | m-chlorobenzoic acid | acetone |
| 25 | 2,4,6,7-tetrachlorophthalic anhydride | acetone |
| 26 | 2-chloropyridine | acetone |
| 27 | 1-(2-ethoxy-2-oxoethyl)pyridinium bromide | water |

Figure S8 depicts the measurement chamber of the CamSeeb 2018 device used for recording the Seebeck coefficient in the temperature range from room temperature to 100^o^C. A CNT film sample, exposed to a temperature gradient, is visible in the chamber.


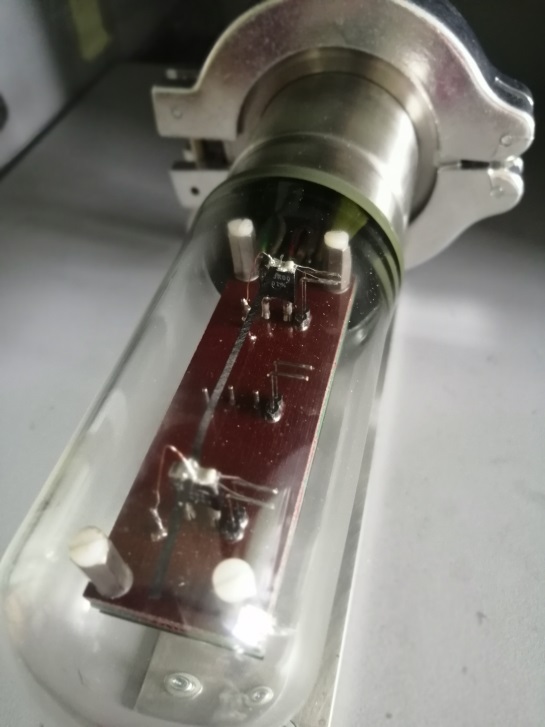


Figure S8 Chamber for determination of Seebeck coefficient of the doped CNT films.


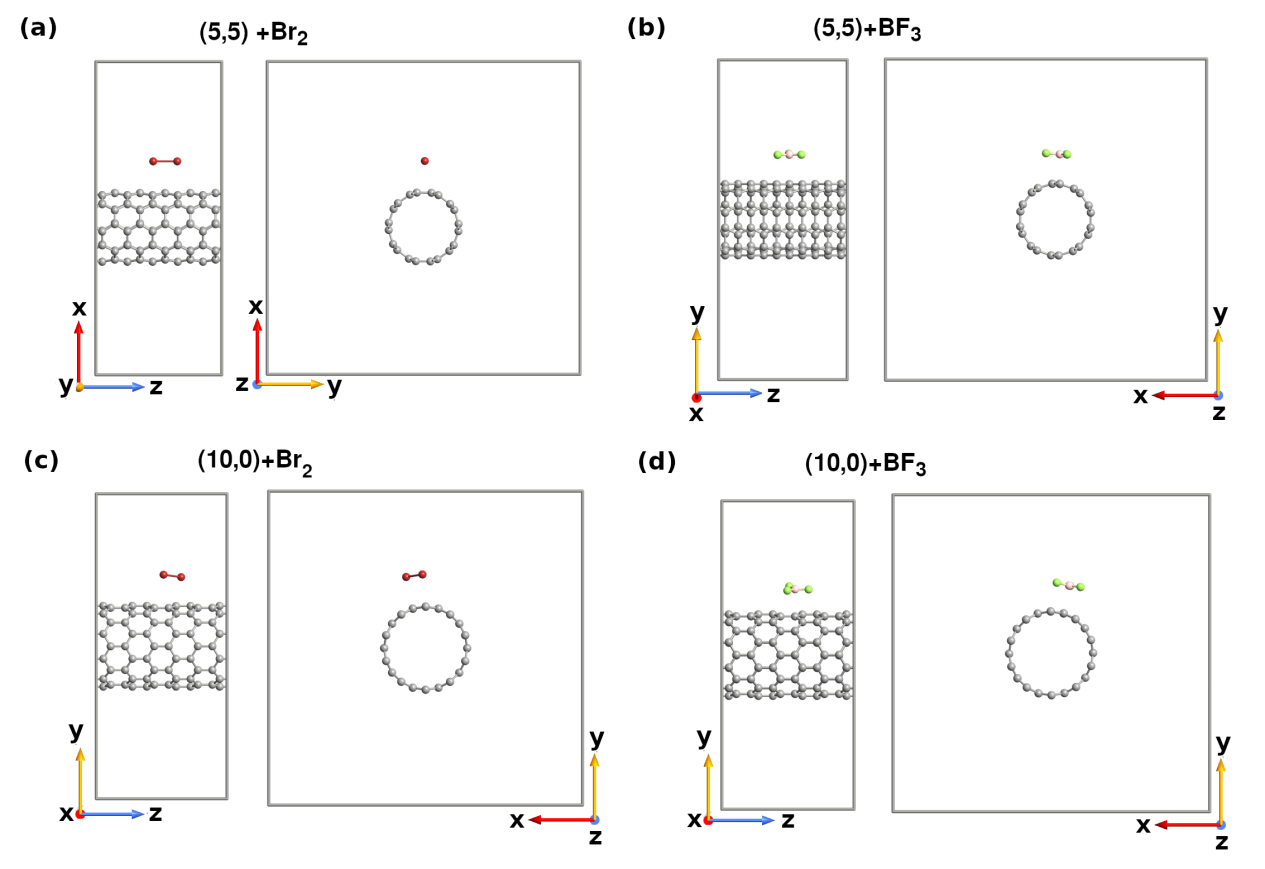


Figure S9 Atomistic cross-sectional and side views of fully optimized (5,5) and (10,0) CNTs interacting with (left) Br_2_ and (right) BF_3_ molecules, respectively. Br atoms are depicted in red while B, F and C atoms are shown in pink, light green, and grey, respectively.

Table S4 Computed structural parameters, adsorption energies per carbon atom (E_ads/NC_), binding energies per atom (E_bind/N_), energy band gaps (E_gap_), Fermi levels (E_F_) and the Fermi level shift with respect to VBT (E_F_-VBT) of fully optimized isolated Br_2_ and BF_3_ molecules, pristine and doped (5,5) and (10,0) CNTs. B-F bond length is calculated as the arithmetic average of three B-F bonds. The Br_2_/BF_3_-CNT equilibrium distance is the mean difference between the molecule atom distances (Br, B, F) from CNT symmetry axis and the mean of the CNT radius. The coefficient of CNT radius variation, CV, is defined as the ratio of the standard deviation to the mean of the CNT radius [10]. The adsorption energy per carbon atom, E_ads/NC_, is calculated as the difference between the total energies of fully optimized systems containing CNT and Br_2_/BF_3_ molecules and the total energies of isolated CNT and Br_2_/BF_3_ molecules corrected using a counterpoise correction [11] and divided by the number of carbon atoms. The binding energy per atom, E_bind/N_, is defined as the difference between the total energies of fully optimized systems (pure or doped CNTs or molecules) and the sum of total atomic energies of the free atom of each type that are present in the systems, divided by the number of all atoms in the system.

| CNT | molecule | Br-Br/B-F bond length [Å] | Br_2_/BF_3_-CNT equilibrium distance [Å] | CV | E_ads/NC_ [eV] | E_bind/N_ [eV] | E_gap,_ [eV] | E_F_ [eV] | E_F_-VBT [eV] |
| --- | --- | --- | --- | --- | --- | --- | --- | --- | --- |
| ---- | Br_2_ | 2.312 | ---- | ---- | ---- | -1.398 | ---- | ---- | ---- |
| ---- | BF_3_ | 1.320 | ---- | ---- | ---- | -5.793 | ---- | ---- | ---- |
| (5,5) | ---- | ---- | ---- | 0.0001 | ---- | -8.162 | 0 | -3.767 | 3.767 |
|  | Br_2_ | 2.365 | 3.039 | 0.0044 | -0.0100 | -8.035 | 0 | -4.348 | 4.348 |
|  | BF_3_ | 1.324 | 2.885 | 0.0044 | -0.0070 | -8.075 | 0.004 | -3.816 | 3.813 |
| (10,0) | ---- | ---- | ---- | 0.0000 | ---- | -8.205 | 0.786 | -3.877 | 3.486 |
|  | Br_2_ | 2.341 | 3.076 | 0.0008 | -0.0090 | -8.098 | 0.490 | -4.081 | 3.886 |
|  | BF_3_ | 1.325 | 2.738 | 0.0014 | -0.0081 | -8.132 | 0.775 | -3.928 | 3.541 |

Table S5 Computed structural parameters, adsorption energies per carbon atom (E_ads.NC_), binding energies per atom (E_bind/N_), energy band gaps (E_gap_) and Fermi levels (E_F_) of fully optimized pristine and doped (12,12) and (20,0) CNTs.

| CNT | molecule | Br-Br/B-F bond length [Å] | Br_2_/BF_3_-CNT equilibrium distance [Å] | CV | E_ads/NC_ [eV] | E_bind/N_ [eV] | E_gap,_ [eV] | E_F,_ [eV] | E_F_-VBT [eV] |
| --- | --- | --- | --- | --- | --- | --- | --- | --- | --- |
| (12,12) | ---- | ---- | ---- | 0.0000 | ---- | -8.301 | 0.000 | -3.970 | 3.971 |
|  | Br_2_ | 2.350 | 3.077 | 0.0019 | -0.0025 | -8.262 | 0.000 | -3.996 | 3.996 |
|  | BF_3_ | 1.324 | 2.753 | 0.0041 | -0.0018 | -8.273 | 0.002 | -3.979 | 3.979 |
| (20,0) | ---- | ---- | ---- | 0.0002 | ---- | -8.299 | 0.470 | -3.957 | 3.725 |
|  | Br_2_ | 2.349 | 3.109 | 0.0004 | -0.0033 | -8.258 | 0.437 | -4.010 | 3.820 |
|  | BF_3_ | 1.325 | 2.704 | 0.0007 | -0.0030 | -8.264 | 0.447 | -3.973 | 3.751 |

References:

1. Puchades, I.; Lawlor, C.C.; Schauerman, C.M.; Bucossi, A.R.; Rossi, J.E.; Cox, N.D.; Landi, B.J. Mechanism of chemical doping in electronic-type-separated single wall carbon nanotubes towards high electrical conductivity. *J. Mater. Chem. C* **2015**, *3*, 10256–10266.

2. Jackson, R.K.; Munro, A.; Nebesny, K.; Armstrong, N.; Graham, S. Evaluation of Transparent Carbon Nanotube Networks of Homogeneous Electronic Type. *ACS Nano* **2010**, *4*, 1377–1384.

3. Behabtu, N.; Young, C.C.; Tsentalovich, D.E.; Kleinerman, O.; Wang, X.; Ma, A.W.K.; Bengio, E.A.; ter Waarbeek, R.F.; de Jong, J.J.; Hoogerwerf, R.E.; et al. Strong, light, multifunctional fibers of carbon nanotubes with ultrahigh conductivity. *Science (80-. ).* **2013**, *339*, 182–6.

4. Fischer, J.E. Chemical doping of single-wall carbon nanotubes. *Acc. Chem. Res.* **2002**, *35*, 1079–1086.

5. Skákalová, V.; Kaiser, A.B.; Dettlaff-Weglikowska, U.; Hrnčariková, K.; Roth, S. Effect of chemical treatment on electrical conductivity, infrared absorption, and raman spectra of single-walled carbon nanotubes. *J. Phys. Chem. B* **2005**, *109*, 7174–7181.

6. Ryu, Y.; Yin, L.; Yu, C. Dramatic electrical conductivity improvement of carbon nanotube networks by simultaneous de-bundling and hole-doping with chlorosulfonic acid. *J. Mater. Chem.* **2012**, *22*, 6959–6964.

7. Nonoguchi, Y.; Ohashi, K.; Kanazawa, R.; Ashiba, K.; Hata, K.; Nakagawa, T.; Adachi, C.; Tanase, T.; Kawai, T. Systematic Conversion of Single Walled Carbon Nanotubes into n-type Thermoelectric Materials by Molecular Dopants. *Sci. Rep.* **2013**, *3*, 3344.

8. Qiu, L.; Zou, H.; Zhu, N.; Feng, Y.; Zhang, X.; Zhang, X. Iodine nanoparticle-enhancing electrical and thermal transport for carbon nanotube fibers. *Appl. Therm. Eng.* **2018**, *141*, 913–920.

9. Zhao, Y.; Wei, J.; Vajtai, R.; Ajayan, P.M.; Barrera, E. V. Iodine doped carbon nanotube cables exceeding specific electrical conductivity of metals. *Sci. Rep.* **2011**, *1*, 83.

10. Milowska, K. Z. Influence of Carboxylation on Structural and Mechanical Properties of Carbon Nanotubes: Composite Reinforcement and Toxicity Reduction Perspectives. *J. Phys. Chem. C*, **2015**, *119*, 26734–26746.

11. Boys, S.; Bernardi, F. The calculation of small molecular interactions by the differences of separate total energies. Some procedures with reduced errors. *Molecular Physics* **1970**, *19*, 553.
